# Supplementary figures and images for: Carotenoid composition and sequestration in cassava (Manihot esculentum Crantz) roots
Source: PLoS One. 2024 Nov 18;19(11):e0312517. doi: 10.1371/journal.pone.0312517 (PMC11573132; doi:10.1371/journal.pone.0312517)

Fig. S1: Chromatograms of lipid analysis of all fractions of GM3736-71 and GM3736-78.

GM3736-71

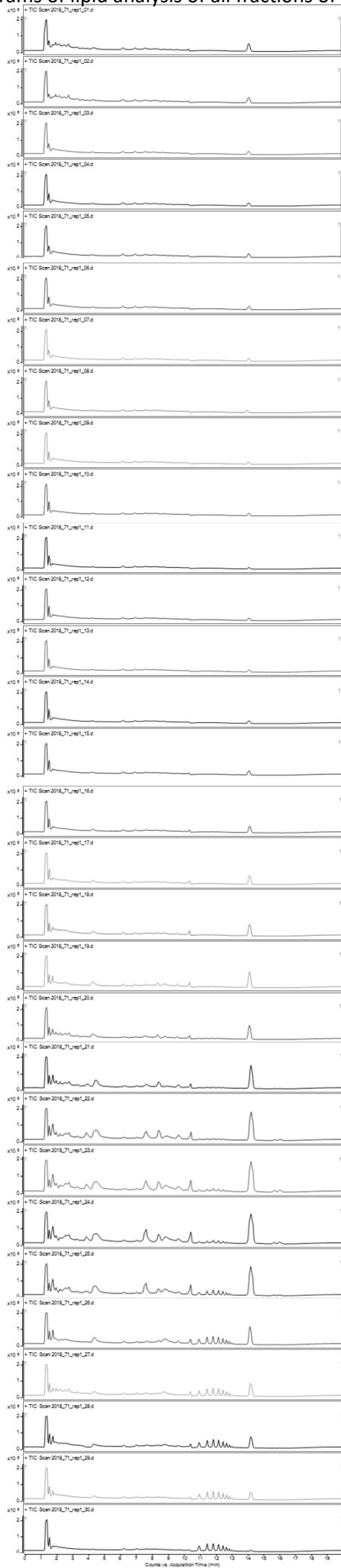

GM3736-78

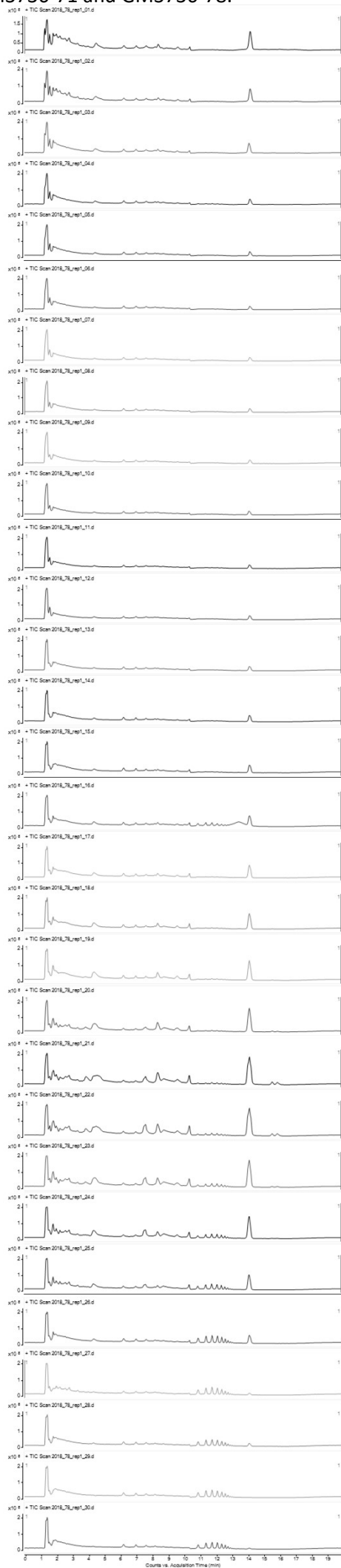

Supplement: S1 Fig — (PDF) [file pone.0312517.s001.pdf]
