# Supplementary material for: Carotenoid composition and sequestration in cassava (Manihot esculentum Crantz) roots
Source: PLoS One. 2024 Nov 18;19(11):e0312517. doi: 10.1371/journal.pone.0312517 (PMC11573132; doi:10.1371/journal.pone.0312517)
Supplement: S2 Fig — Correlation analysis of β-carotene to glucose, sucrose and dry matter of breeding populations (A) GM3732/3736 in 2020, (B) GM5309 and (C) GM5270. (PDF) [file pone.0312517.s002.pdf]

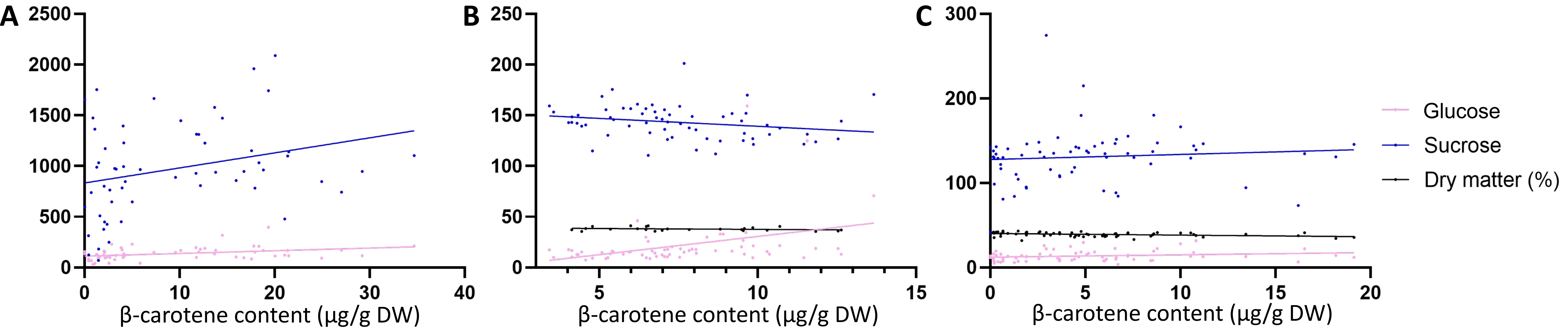

	R^2	*P*-value
Glucose	0.1315	0.0287
Sucrose	0.0826	0.0051

	R^2	*P*-value
Glucose	0.1292	0.0038
Sucrose	0.5226	NS
Dry matter	0.6650	NS

	R^2	*P*-value
Glucose	0.0414	NS
Sucrose	0.0072	NS
Dry matter	0.1288	0.0039

Fig. S2. Correlation analysis of β-carotene to glucose, sucrose and dry matter of breeding populations (A) GM3732/3736 in 2020, (B) GM5309 and (C) GM5270. Results of the correlation are visualised at the top and coefficient of determination (R^2) and significance (*P*-value) are listed below.
