# Supplementary material for: Carotenoid composition and sequestration in cassava (Manihot esculentum Crantz) roots
Source: PLoS One. 2024 Nov 18;19(11):e0312517. doi: 10.1371/journal.pone.0312517 (PMC11573132; doi:10.1371/journal.pone.0312517)
Supplement: S1 Table — Breeding strategy for GM3732 and GM3736 is displayed as an ancestral tree and contribution of parental lines to GM5270 and GM5309 is listed as percentage. (DOCX) [file pone.0312517.s003.docx]

Table S1. Parental background for cassava carotenoid populations studied. Breeding strategy for GM3732 and GM3736 is displayed as an ancestral tree and contribution of parental lines to GM5270 and GM5309 is listed as percentage.

| Population | Breeding strategy / percentage of parental lines | | | | | | | | | |
| --- | --- | --- | --- | --- | --- | --- | --- | --- | --- | --- |
| GM3736  GM3732 | 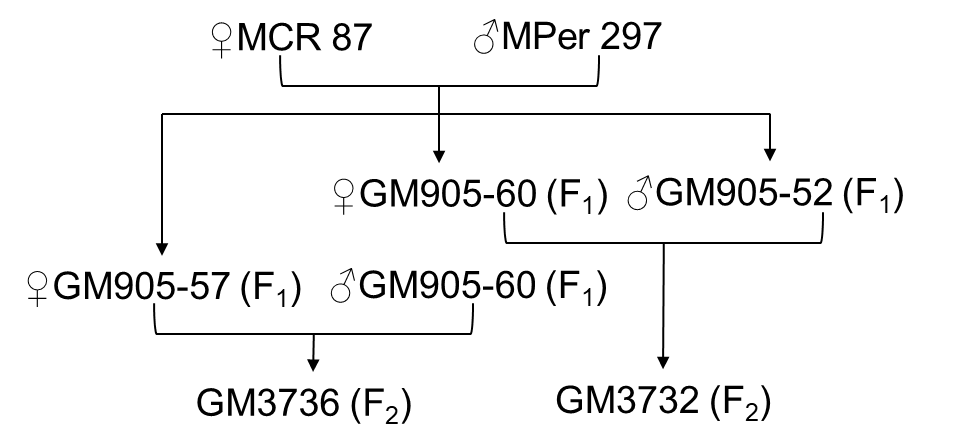 | | | | | | | | | |
|  |  | | | | | | | | | |
|  | MAL66 | BRA1A | PER297 | VEN185 | PAN70 | COL638 | COL1684 | COL647 | COL2295 | COL1734 |
| GM5270 | 18.75% | 25% | 18.75% | 18.75% | 1.6% | 1.6% | 1.6% | 1.6% | 6.25% | 6.25% |
| GM5309 | 25% | 25% | 18.75% | 18.75% | 1.6% | 1.6% | 1.6% | 1.6% | 6.25% |  |
