# Supplementary material for: Carotenoid composition and sequestration in cassava (Manihot esculentum Crantz) roots
Source: PLoS One. 2024 Nov 18;19(11):e0312517. doi: 10.1371/journal.pone.0312517 (PMC11573132; doi:10.1371/journal.pone.0312517)
Supplement: S11 Table — (PDF) [file pone.0312517.s013.pdf]

Fraction	Database code	Protein name	Protein MW	Protein size (kDa)	Score	Coverage (%)	No. of matched peptides	Spectrum Mill			Mascot			Peptide sequence	Cellular compartment	Biological function
								L	M	H	L	M	H			
1	Q1AP39	14-3-3 protein	1418.70	29.83	14.03	47.7	16		x			x		TVDVEELTVEER	cytoplasm	Transport
1	A0A2C9VXB8	Aamy domain-containing protein	1422.67	96.69	18.17 - 20.73	55.9	54	x	x	x		x	x	VALDSDAWEFGGR	cytoplasm/plastid	Carbohydrate metabolism
1	A0A2C9WDD9	Alpha-1,4 glucan phosphorylase	2154.11	107.53	11.68 - 18.94	59.3	61		x	x			x	FITDVGATVNHDPEIGDLLK	cytoplasm	Carbohydrate metabolism
1	A0A251J147	Aspartate aminotransferase	1508.77	50.80	10.09	10.3	5		x			x		TVGLDFEGMIADIK	plastid	Nitrogen/sulphur metabolism
1	A0A2C9VPN2	CYTOSOL_AP domain-containing protein	1553.88	60.99	16.3	36.7	20		x			x		SLDILGLGIGPEIEK	cytoplasm/plastid	remove peptides
1	A0A2C9UC96	DUF3700 domain-containing protein	1448.80	25.38	14.23	23.7	5		x			x		SANEVILVIEAYK	unknown	Nitrogen/sulphur metabolism
1	A0A251J2Y7	Elongation factor 1-alpha	1025.61	49.38	10.89	21.1	12		x			x		IGGIGTVPVGR	organelle membrane	translation
1	A0A2C9U930	Formate dehydrogenase, mitochondrial	955.57	42.29	12.86	30.3	13		x			x		GVLIVNNAR	plastid	Stress related
1	A0A251IW93	GLOBIN domain-containing protein	975.55	18.31	14.1	39.5	6		x			x		IFEIAPSAK	unknown	Oxygen carrier
1	A0A2C9UK69	Glyceraldehyde-3-phosphate dehydrogenase	1291.61	36.86	9.41 - 12.31	71.8	26		x	x			x	TVDGPSMKDWR	cytoplasm/plastid	Carbohydrate metabolism
1	A0A251J164	GTP-binding nuclear protein	1228.62	25.11	11.08 - 13.39	21.2	6		x	x		x	x	NLQYYEISAK	cytoplasm/nucleus	Transport
1	A0A2C9VRW1	Phosphoglycerate kinase	1085.66	42.39	20.85	45.1	15		x			x		LSELLGVQVK	plastid	Carbohydrate metabolism
1	V9M4W7	Plastid 16-kDa outer membrane protein	1243.67	15.70	15.45 - 16.99	29.4	7		x	x		x		VLAEDAYHVVK	chloroplast membrane	Transport
1	A0A2C9V3S4	SHSP domain-containing protein	901.51	18.76	16.56 - 16.9	67.2	26		x	x	x		x	VLQISGER	UNK	Protein/Stress related
1	A0A2C9WHT3	SHSP domain-containing protein	1336.66	26.17	12.7 - 21.75	63.6	29	x	x	x		x	x	IFEDAMTLPGSR	unknown	Stress related
1	A0A076NB98	Sucrose synthase	1268.73	92.59	14.28 - 17.65	54	44		x	x		x	x	IKQQGLDITPR	plastid	Carbohydrate metabolism
1	A0A2C9UDR8	TCTP domain-containing protein	1063.58	19.04	12.32	14.2	3		x			x		VVDIVDTFR	cytoplasm	Cell proliferation and differentiation
1	A0A251JQ24	Uncharacterized protein	1954.06	41.70	13.55 - 18.17	65.2	27		x	x	x	x	x	VAPEEHPVLLTEAPLNPK	cytoplasm	Transport
1	A0A199U950	Uncharacterized protein (Fragment)	1021.53	36.60	6.91 - 14.66	48.3	15		x	x			x	DTDILAAFR	chloroplast	Carbohydrate metabolism
20	A0A2C9VXB8	Aamy domain-containing protein	1422.67	96.69	18.8 - 23.72	55.9	54	x	x	x	x	x	x	VALDSDAWEFGGR	cytoplasm/plastid	Carbohydrate metabolism
20	A0A2C9WDD9	Alpha-1,4 glucan phosphorylase	1885.89	107.53	12.73 - 19.74	59.3	61	x	x	x	x	x	x	APAEDLDLSAFNAGEHTK	cytoplasm	Carbohydrate metabolism
20	A0A2C9VN82	Ferritin	1142.65	29.37	13.14	48.4	11			x			x	LLNLQSVAER	cytoplasm/plastid	Transport
20	V9M4W7	Plastid 16-kDa outer membrane protein	1243.67	15.70	12.72 - 16.14	29.4	7	x	x	x	x	x	x	VLAEDAYHVVK	chloroplast membrane	Transport
20	A0A2C9V3S4	SHSP domain-containing protein	1510.71	18.76	18.81 - 22.25	67.2	26	x		x	x		x	SELANETSAFANTR	UNK	Protein/Stress related
20	A0A2C9WHT3	SHSP domain-containing protein	1289.74	26.17	9.92 - 21.61	63.6	29	x	x	x		x	x	VFVEEDVLVIK	unknown	Stress related
20	A0A076NB98	Sucrose synthase	1225.66	92.59	11.04 - 14.26	54	44	x	x	x	x		x	VVHGIDVFDPK	plastid	Carbohydrate metabolism
20	A0A199U950	Uncharacterized protein (Fragment)	1021.53	36.60	14.16	48.3	15			x			x	DTDILAAFR	chloroplast	Carbohydrate metabolism
21	A0A2C9WFF9	14_3_3 domain-containing protein	1772.97	28.50	23.95	69.5	19			x			x	AAQDIALADLAPTHPIR	cytoplasm	Transport
21	A0A2C9UIY2	40S ribosomal protein SA	940.58	34.19	11.5	14.1	5			x		x	x	LLILTDPR	cytoplasm	translation
21	A0A2C9VXB8	Aamy domain-containing protein	1422.67	96.69	20.19 - 24.06	55.9	54	x	x	x	x	x	x	VALDSDAWEFGGR	cytoplasm/plastid	Carbohydrate metabolism
21	A0A2C9WDD9	Alpha-1,4 glucan phosphorylase	2154.11	107.53	17.32 - 24.53	59.3	61	x	x	x	x	x	x	FITDVGATVNHDPEIGDLLK	cytoplasm	Carbohydrate metabolism
21	Q52QX1	Ascorbate peroxidase APX2	2094.88	27.67	20.26 - 26.66	60.4	16		x	x			x	YAADEEAFFADYAESHMK	chloroplast	Stress related
21	A0A251J147	Aspartate aminotransferase	1508.77	50.80	17.72	10.3	5			x			x	TVGLDFEGMIADIK	plastid	Nitrogen/sulphur metabolism
21	A0A2C9UKP7	ATP synthase subunit beta	2186.15	60.46	13.99	37	15			x			x	IPSAVGYQPTLATDLGGLQER	organelle membrane	Transport
21	A0A2C9VVU3	Catalase domain-containing protein	2732.25	57.34	19.23 - 21.46	46.9	22	x	x	x	x	x	x	LFIQTMDPADEDKFDFDPLDMTK	cytoplasm/plasma membrane	Stress related
21	A0A2C9VN82	Ferritin	1142.65	29.37	18.02	48.4	11			x			x	LLNLQSVAER	cytoplasm/plastid	Transport
21	A0A2C9U930	Formate dehydrogenase, mitochondrial	2675.32	42.29	16.7	30.3	13			x			x	AAADAGLTVAEVTGSNVVSVAEDELMR	plastid	Stress related
21	A0A2C9UU97	Glucose-1-phosphate adenylyltransferase	1048.54	57.33	15.45	46.8	27			x			x	SSPIYTQPR	chloroplast	Carbohydrate metabolism
21	A0A2C9TZL8	HATPase_c domain-containing protein	1071.58	93.19	11.27	7.7	6			x			x	LGIIEDAANR	chloroplast stroma/ER/cytoplasm/plasma membrane	Protein related
21	A0A251LEJ6	Histone H4	1180.62	11.41	16.05	50.4	5			x			x	ISGLIYEETR	nucleus	Nucleic acid related
21	A0A2C9UCS2	Malate dehydrogenase	1346.74	35.80	17.43	57.8	16			x			x	MELVDAAFPLLK	plastid	Carbohydrate metabolism
21	A0A2C9UYF6	Phospholipase D	1146.60	91.91	13.27	35	21			x		x	x	VLMLVWDDR	organelle membrane	Lipid metabolism
21	A0A251LKW3	PKS_ER domain-containing protein	1018.58	39.08	10.99 - 16.03	15.7	5	x		x	x	x	x	VTVISTSPSK	cytoplasm	Lignin biosynthesis
21	V9M4W7	Plastid 16-kDa outer membrane protein	1399.77	15.70	9.02 - 20.99	29.4	7	x	x	x		x	x	VLAEDAYHVVKR	chloroplast membrane	Transport
21	A0A2C9WHT3	SHSP domain-containing protein	1289.74	26.17	17.75 - 23.18	63.6	29		x	x			x	VFVEEDVLVIK	unknown	Stress related
21	A0A076NB98	Sucrose synthase	1273.65	92.59	13.46 - 18.58	54	44	x	x	x			x	VFGTEHSDILR	plastid	Carbohydrate metabolism
21	A0A2C9UL58	Tubulin beta chain	1139.69	49.84	12.37	21.3	9			x			x	LAVNLIPFPR	plastid	Cytoskeleton/division
21	A0A251JQ24	Uncharacterized protein	1774.90	41.70	14.89 - 23	65.2	27	x	x	x		x	x	NYELPDGQVITIGAER	cytoplasm	Transport
21	A0A199U950	Uncharacterized protein (Fragment)	1021.53	36.60	14.76 - 19.82	48.3	15		x	x		x	x	DTDILAAFR	chloroplast	Carbohydrate metabolism
22	A0A2C9WFF9	14_3_3 domain-containing protein	1772.97	28.50	18.58 - 26.13	69.5	19	x	x	x	x	x	x	AAQDIALADLAPTHPIR	cytoplasm	Transport
22	Q1AP39	14-3-3 protein	1788.96	29.83	12.78 - 18.65	47.7	16	x	x	x		x	x	SAQDIALADLAPTHPIR	cytoplasm	Transport
22	A0A2C9UIY2	40S ribosomal protein SA	940.58	34.19	16.27	14.1	5		x		x	x		LLILTDPR	cytoplasm	translation
22	A0A2C9V405	60S acidic ribosomal protein P0	1411.84	34.15	13.43	28.1	7	x				x		GTVEIITPVELIK	cytoplasm	translation
22	A0A2C9VXB8	Aamy domain-containing protein	1286.60	96.69	20.45 - 21.97	55.9	54	x	x	x			x	SVSEGGIGFDYR	cytoplasm/plastid	Carbohydrate metabolism
22	A0A2C9W1S2	ADP,ATP carrier protein	1256.67	67.60	14.46	8.8	6	x			x			AAIDVVCNPLGK	chloroplast membrane	Transport
22	A0A251KRD9	AIG1-type G domain-containing protein	805.48	26.07	11.02	7.6	3		x			x		LVELFGK	chloroplast	Transport
22	A0A2C9WDD9	Alpha-1,4 glucan phosphorylase	1885.89	107.53	19.88 - 22.17	59.3	61	x	x	x	x	x	x	APAEDLDLSAFNAGEHTK	cytoplasm	Carbohydrate metabolism
22	Q52QX1	Ascorbate peroxidase APX2	2094.88	27.67	20.61 - 27.77	60.4	16	x	x	x	x	x	x	YAADEEAFFADYAESHMK	chloroplast	Stress related
22	A0A2C9VVU3	Catalase domain-containing protein	2732.25	57.34	21.88 - 22.98	46.9	22	x	x		x	x		LFIQTMDPADEDKFDFDPLDMTK	cytoplasm/plasma membrane	Stress related
22	A0A2C9UI66	Cytochrome b5 heme-binding domain-containing protein	1166.58	22.98	12.88	4.7	1			x			x	GQIYDISQSR	membrane	Membrane protein
22	A0A2C9UC96	DUF3700 domain-containing protein	1448.80	25.38	15.11	23.7	5		x			x		SANEVILVIEAYK	unknown	Nitrogen/sulphur metabolism
22	A0A2C9VN82	Ferritin	1142.65	29.37	13.14 - 18.31	48.4	11		x	x		x	x	LLNLQSVAER	cytoplasm/plastid	Transport
22	A0A251IW93	GLOBIN domain-containing protein	975.55	18.31	12.53	39.5	6			x			x	IFEIAPSAK	unknown	Oxygen carrier
22	A0A2C9UK69	Glyceraldehyde-3-phosphate dehydrogenase	1305.65	36.86	15.3 - 15.78	71.8	26	x	x		x	x		DAPMFVVGVNEK	cytoplasm/plastid	Carbohydrate metabolism
22	A0A251J164	GTP-binding nuclear protein	1015.58	25.11	12	21.2	6		x		x	x		LVIVGDGGTGK	cytoplasm/nucleus	Transport
22	A0A251LEJ6	Histone H4	1180.62	11.41	18.43	50.4	5			x	x		x	ISGLIYEETR	nucleus	Nucleic acid related
22	A0A2C9UCS2	Malate dehydrogenase	1346.74	35.80	17.54 - 20.36	57.8	16	x	x		x	x		MELVDAAFPLLK	plastid	Carbohydrate metabolism
22	A0A2C9W0Z0	MlaD domain-containing protein	1507.95	41.68	11.46 - 15.76	4.9	2	x	x		x			VAAIIEEAKPLLLK	chloroplast membrane	Lipid metabolism
22	A0A2C9TZM7	Peptidyl-prolyl cis-trans isomerase	1392.72	18.11	9.08	40.6	8			x			x	IVMELFADTTPR	cytoplasm	Protein related
22	A0A2C9UAL9	Peroxiredoxin	1033.59	17.33	12.93 - 18.04	31.4	7		x	x		x	x	FALLVDDLK	cytoplasm	Stress related
22	A0A2C9UYF6	Phospholipase D	1146.60	91.91	14.33 - 17.7	35	21	x	x		x	x		VLMLVWDDR	organelle membrane	Lipid metabolism
22	A0A199UAY8	Plasma membrane-associated cation-binding protein 1	1164.63	22.46	13.17	15.6	4			x			x	VSTFVVVEER	membrane	Lipid related
22	V9M4W7	Plastid 16-kDa outer membrane protein	1399.77	15.70	19.14 - 24.46	29.4	7	x	x	x	x	x	x	VLAEDAYHVVKR	chloroplast membrane	Transport
22	A0A2C9UQH2	PLAT domain-containing protein	1235.63	19.32	17.01	20.3	4			x			x	DASPYELTAIR	membrane	Signalling
22	A0A2C9WA12	Reticulon-like protein	1275.71	28.77	16.18	18.6	5			x			x	VEINQAFSVLR	ER (membrane)	Stress related
22	A0A2C9VVU2	Ribosomal_L18_c domain-containing protein	955.59	23.95	9.68	19.9	5		x			x		ALLDVGLVR	cytoplasm	translation
22	A0A2C9U6Q7	RRM domain-containing protein	1232.60	16.36	18.99	23.6	5			x			x	GFGFVTFSNEK	unknown	transcription
22	A0A2C9VZB5	SAM domain-containing protein	1813.01	27.83	11.83 - 15.64	22.3	7	x	x		x	x		GLLTDNTLPLLTDSALR	organelle membrane	protein insertion into mitochondrial inner membrane [GO:0045039]; protein targeting to chloroplast [GO:0045036]
22	A0A2C9WHT3	SHSP domain-containing protein	1336.66	26.17	20.06 - 23.87	63.6	29	x	x	x	x	x	x	IFEDAMTLPGSR	unknown	Stress related
22	A0A076NB98	Sucrose synthase	2482.19	92.59	13.29 - 19.93	54	44	x	x	x	x	x	x	KAEEYLTALSPDTPYSQFEHR	plastid	Carbohydrate metabolism
22	A0A2C9VLE6	Superoxide dismutase	2019.01	25.87	19.2	39	9			x			x	ALEQLNDAMEKGDSATVVK	mitochondria	Stress related
22	A0A251JQ24	Uncharacterized protein	1855.93	41.70	17.33 - 23.5	65.2	27	x	x	x	x	x	x	LAYVALDYEQELETAK	cytoplasm	Transport

Fraction	Database code	Protein name	Protein MW	Protein size (kDa)	Score	Coverage (%)	No. of matched peptides	Spectrum Mill			Mascot			Peptide sequence	Cellular compartment	Biological function
								L	M	H	L	M	H			
22	A0A199U950	Uncharacterized protein (Fragment)	1465.76	36.60	15.16 - 18.4	48.3	15	x	x	x	x	x	x	TFQGPPHGIQVER	chloroplast	Carbohydrate metabolism
22	A0A251LGJ0	UTP--glucose-1-phosphate uridylyltransferase	2081.09	51.50	17.17	64.1	26		x		x	x		VQLLEIAQVPDEHVSEFK	cytoplasm	Carbohydrate metabolism
23	A0A2C9WFF9	14_3_3 domain-containing protein	1772.97	28.50	19.9 - 23.61	69.5	19	x	x	x			x	AAQDIALADLAPTHPIR	cytoplasm	Transport
23	Q1AP39	14-3-3 protein	1418.70	29.83	19.1 - 21.37	47.7	16	x	x	x			x	TVDVEELTVEER	cytoplasm	Transport
23	A0A251K8D8	40S ribosomal protein S24	1080.57	15.68	16.86	40.1	7	x			x			DPNTIFVFK	cytoplasm	translation
23	A0A2C9UZC1	40S ribosomal protein S25	1114.65	12.05	13.15 - 15.57	20.3	3	x	x	x	x	x	x	LITPSILSDR	cytoplasm	translation
23	A0A2C9VGW5	40S ribosomal protein S4	2117.01	29.89	15.06 - 18.36	56.2	17	x	x	x	x	x	x	GSFETIHIQDATGHEFATR	cytoplasm	translation
23	A0A2C9UTK8	40S ribosomal protein S7	900.55	22.09	11.97 - 16.28	24.6	4	x	x		x	x		DVILIATR	cytoplasm	translation
23	A0A2C9UDR1	40S ribosomal protein S8	1718.91	24.86	15.29 - 19.81	22.3	6	x	x	x	x	x	x	ILDVVYNASNNELVR	cytoplasm	translation
23	A0A2C9UIY2	40S ribosomal protein SA	940.58	34.19	12.48	14.1	5			x	x	x	x	LLILTDPR	cytoplasm	translation
23	A0A2C9V405	60S acidic ribosomal protein P0	1411.84	34.15	13.45 - 15.28	28.1	7	x	x	x	x	x	x	GTVEIITPVELIK	cytoplasm	translation
23	A0A2C9VGW4	60S ribosomal protein L13	922.49	23.69	10.99 - 12.29	34.9	7		x	x			x	GFSLEELK	cytoplasm	translation
23	A0A2C9W9Y7	6-phosphogluconate dehydrogenase, decarboxylating	1641.86	54.20	11.34	7.8	5		x			x		NPNLASLVVDPEFAR	cytoplasm	Carbohydrate metabolism
23	A0A2C9VYT5	AAA domain-containing protein	1934.99	44.67	11.8 - 13.7	10.2	3	x	x		x	x		TLMELLNQLDGFDQLGK	cytoplasm/nucleus	transcription
23	A0A2C9VXB8	Aamy domain-containing protein	1286.60	96.69	21.17 - 23.41	55.9	54	x	x	x	x	x	x	SVSEGGIGFDYR	cytoplasm/plastid	Carbohydrate metabolism
23	A0A2C9WDD9	Alpha-1,4 glucan phosphorylase	1556.68	107.53	21.63 - 24.78	59.3	61	x	x	x	x	x	x	FADNEDFQTQWR	cytoplasm	Carbohydrate metabolism
23	A0A2C9U177	Annexin	1403.75	36.03	13.05 - 19.18	62.6	20	x	x	x	x	x	x	ADPKDEFLSLLR	cytoplasm	Stress related
23	Q52QX1	Ascorbate peroxidase APX2	2094.88	27.67	23.86 - 26.62	60.4	16	x	x	x	x	x	x	YAADEEAFFADYAESHMK	chloroplast	Stress related
23	A0A2C9W3S2	Aspartate aminotransferase	1453.84	44.49	11.42 - 11.42	16.4	7	x	x	x	x	x		TEEGKPLVLNVVR	plastid	Nitrogen/sulphur metabolism
23	A0A2C9UKP7	ATP synthase subunit beta	1457.84	60.46	20.67 - 22.63	37	15	x	x	x	x	x	x	TVLIMELINNVAK	organelle membrane	Transport
23	A0A2C9VFD2	Calreticulin	1234.68	49.45	14.72 - 16.59	6.6	3	x	x	x	x			YVGIELWQVK	ER (membrane)	Protein related
23	Q9SW99	Catalase CAT1	1557.80	57.17	20.02 - 21.83	35.5	19	x	x	x	x	x		GPILLEDYHMIEK	cytoplasm/plasma membrane	Stress related
23	A0A2C9VVU3	Catalase domain-containing protein	1557.80	57.34	20.02 - 21.83	46.9	22	x	x	x	x	x	x	GPILLEDYHMIEK	cytoplasm/plasma membrane	Stress related
23	A0A2C9V3F3	Clathrin heavy chain	1970.03	193.09	15.86 - 16.97	3.1	5	x	x		x	x		AFMTADLPHELIELLEK	Golgi apparatus	Transport
23	A0A2C9UI66	Cytochrome b5 heme-binding domain-containing protein	1166.58	22.98	13.82	4.7	1		x			x		GQIYDISQSR	membrane	Membrane protein
23	A0A2C9UBZ0	Cytochrome b-c1 complex subunit 7	1230.68	14.71	16.41	9	1	x			x			EALGALPLYQR	mitochondria	Transport
23	A0A2C9U871	Cytochrome c oxidase subunit 5C	1283.66	6.88	12.42	15.6	1		x			x		AKEFYDLLER	organelle membrane	UNK
23	A0A2C9VPN2	CYTOSOL_AP domain-containing protein	2152.15	60.99	16.18 - 18.4	36.7	20	x	x	x	x	x	x	ELVNSPANVLTPAVLAEEASK	cytoplasm/plastid	remove peptides
23	A0A2C9V419	D-3-phosphoglycerate dehydrogenase	1129.61	62.93	14.2 - 15.42	19.6	9	x	x	x	x	x		GGVIDEEALVK	chloroplast stroma	Nitrogen/sulphur metabolism
23	V9M4V2	Delta-1-pyrroline-5-carboxylate synthase	2327.21	80.14	18.84 - 19.08	5.6	3	x	x		x			ADLLILLSDVEGLYSGPPSDPR	cytoplasm	Amino acid synthesis
23	A0A2C9URR0	Dolichyl-diphosphooligosaccharide--protein glycosyltransferase 48 kDa subunit	1275.76	48.79	11.92	6.4	3		x		x			ILVLLDDFSLK	organelle membrane	Protein related
23	A0A2C9UC96	DUF3700 domain-containing protein	1448.80	25.38	13.99 - 18.47	23.7	5	x	x		x	x	x	SANEVILVIEAYK	unknown	Nitrogen/sulphur metabolism
23	A0A2C9WM64	EF1_GNE domain-containing protein	1374.71	23.91	11.83 - 13.92	22.7	4		x	x		x	x	SVEMPGLLWGASK	cytoplasm	translation
23	A0A251J2Y7	Elongation factor 1-alpha	2220.16	49.38	10.97 - 14.33	21.1	12	x	x	x	x	x		MIPTKPMVVETFSGYPPLGR	organelle membrane	translation
23	A0A2C9WKS1	FabA domain-containing protein	1006.57	25.51	10.28	3.4	1			x	x			FPFLLVDR	cytoplasm	Lipid metabolism
23	A0A2C9UPJ8	FAS1 domain-containing protein	1192.66	34.64	14.37	17.9	5			x			x	AFSDLLIASGAK	membrane	Membrane protein
23	A0A2C9VN82	Ferritin	1078.59	29.37	13.09 - 20.91	48.4	11	x	x	x	x	x	x	ISEYVAQLR	cytoplasm/plastid	Transport
23	A0A2C9U930	Formate dehydrogenase, mitochondrial	2675.32	42.29	18.66 - 20.01	30.3	13	x	x	x	x	x	x	AAADAGLTVAEVTGSNVVSVAEDELMR	plastid	Stress related
23	A0A2C9VH59	Fructose-bisphosphate aldolase	3071.57	38.59	17.07 - 18.07	58.1	20	x	x	x	x	x	x	TVPAAVPAIVFLSGGQSEEEATLNLNAMNK	cytoplasm	Carbohydrate metabolism
23	A0A251IW93	GLOBIN domain-containing protein	1391.74	18.31	13.88 - 21.81	39.5	6	x	x	x	x	x	x	VFTEEQEALVVK	unknown	Oxygen carrier
23	A0A2C9UU97	Glucose-1-phosphate adenylyltransferase	1874.92	57.33	13.53 - 17.94	46.8	27	x	x	x	x	x	x	ETDADITVAALPMDEKR	chloroplast	Carbohydrate metabolism
23	A0A2C9UHE4	Glucose-6-phosphate 1-dehydrogenase	1196.74	59.16	11.37 - 15.41	8.3	5	x	x		x	x		ELVQNLLVLR	unknown	Carbohydrate metabolism
23	A0A2C9U0T4	Glucose-6-phosphate isomerase	2042.10	67.85	12.06	13.1	7			x			x	TSEMSAVGLLPAALQGIDIR	cytoplasm	Carbohydrate metabolism
23	A0A2C9UHS4	Glutaredoxin domain-containing protein	1295.79	11.30	17.56 - 20.85	21.4	2	x	x	x	x		x	LIPLLTEAGAVAK	cytoplasm	Stress related
23	A0A2C9UK69	Glyceraldehyde-3-phosphate dehydrogenase	1869.07	36.86	15.36 - 18.13	71.8	26	x	x	x	x	x		VPTVDVSVVDLTVRLEK	cytoplasm/plastid	Carbohydrate metabolism
23	A0A2C9VF59	GRAM domain-containing protein	1298.66	29.85	12.8	4.4	1			x			x	AITEGGFESLFK	membrane	Transport
23	A0A251J164	GTP-binding nuclear protein	1015.58	25.11	12.71	21.2	6		x		x	x	x	LVIVGDGGTGK	cytoplasm/nucleus	Transport
23	A0A2C9V136	Histone H2A	944.53	15.81	10.5	15.4	3	x				x		AGLQFPVGR	nucleus/plastid	Nucleic acid related
23	A0A199UC09	Histone H2B	1180.63	15.40	12.5	45.3	7		x		x	x	x	QVHPDIGISSK	nucleus/plastid	Nucleic acid related
23	A0A251LEJ6	Histone H4	1180.62	11.41	17.99 - 22	50.4	5	x	x	x	x	x	x	ISGLIYEETR	nucleus	Nucleic acid related
23	A0A2C9UAF1	KH type-2 domain-containing protein	1423.67	26.79	14.9 - 18.86	44.8	13	x	x	x		x	x	ELAEDGYSGVEVR	cytoplasm/nucleus	translation
23	A0A2C9UR13	KOW domain-containing protein	1316.72	16.81	14.25	28	6			x	x	x	x	ILMSAPLSTDLR	cytoplasm	translation
23	A0A251KYX0	Late embryogenesis abundant protein 14-A	3497.68	34.83	14.05 - 24.01	35.8	7	x	x	x	x	x	x	LENMNDFDLGLNGLDYEVWLSDVSIGGAELAK	unknown	Stress related
23	A0A2C9UCS2	Malate dehydrogenase	1346.74	35.80	18.72 - 23.59	57.8	16	x	x	x	x	x	x	MELVDAAFPLLK	plastid	Carbohydrate metabolism
23	A0A2C9UAX3	Malic enzyme	2714.43	65.00	15.39 - 21.28	48.3	28	x	x	x	x	x	x	LLIDNVEELLPIVYTPTVGEACQK	chloroplast	Carbohydrate metabolism
23	A0A2C9UPS6	NTF2 domain-containing protein	1733.79	13.60	13.69	34.9	3		x			x		AFVEHYYTTFDANR	cytoplasm	Transport
23	A0A2C9VV21	Nucleoside diphosphate kinase	979.48	16.28	12.65 - 17.18	45.9	9	x	x	x	x	x	x	GDYAIDIGR	plastid/cytoplasm	Nucleic acid related
23	A0A251KVM8	PCI domain-containing protein	1370.81	47.15	13.04 - 16.68	6.1	3	x	x		x	x		LLLVDIDLLESK	cytoplasm	Protein related
23	A0A251J7N7	Pectin acetylesterase	1557.73	43.32	16.15 - 16.44	26	11	x	x		x	x		DVAGGFEIENFFGR	extracellular region	Cytoskeleton/division
23	A0A2C9W531	Peptidase A1 domain-containing protein	1088.65	46.04	14.6 - 17.68	27.6	10		x	x	x	x	x	LVAPVYTAVR	membrane	Stress-related
23	A0A2C9V0Y6	Peptidyl-prolyl cis-trans isomerase	1624.91	17.91	10.99 - 16.08	46.1	10	x	x	x	x	x	x	HVVFGQVVEGLDVVK	cytoplasm	Protein related
23	A0A2C9UAL9	Peroxiredoxin	1033.59	17.33	16.68 - 18.53	31.4	7	x	x	x	x	x	x	FALLVDDLK	cytoplasm	Stress related
23	A0A2C9VRW1	Phosphoglycerate kinase	1903.97	42.39	15.12 - 19.9	45.1	15	x	x		x	x		LAALADVYVNDAFGTAHR	plastid	Carbohydrate metabolism
23	A0A2C9UYF6	Phospholipase D	1933.98	91.91	17.9 - 20.46	35	21	x	x	x	x	x	x	LEGPIAWDVLFNFEQR	organelle membrane	Lipid metabolism
23	A0A2C9WFJ6	Phosphoserine aminotransferase	1067.59	46.77	15.15	14.8	8		x			x		FGLIYAGAQK	cytoplasm	Nitrogen/sulphur metabolism
23	A0A2C9WJ97	PKS_ER domain-containing protein	1192.67	38.62	10.1 - 13.91	30.6	8	x	x	x	x	x		FLDFVVPSIR	UNK	Lignin biosynthesis
23	A0A199UAY8	Plasma membrane-associated cation-binding protein 1	1164.63	22.46	15.82	15.6	4			x			x	VSTFVVVEER	membrane	Lipid related
23	V9M4W7	Plastid 16-kDa outer membrane protein	1243.67	15.70	19.69 - 22.64	29.4	7	x	x	x	x	x	x	VLAEDAYHVVK	chloroplast membrane	Transport
23	A0A2C9UQH2	PLAT domain-containing protein	1235.63	19.32	17.66 - 23.33	20.3	4	x	x	x	x	x	x	DASPYELTAIR	membrane	Signalling
23	A0A2C9VXF7	Pribosyltran domain-containing protein	1271.74	28.44	12.53	14	4	x				x		LGDKPLFVLVNG	cytoplasm	Nucleic acid related
23	A0A2C9UBP1	Profilin	1106.57	14.10	12.67 - 13.59	23.6	3	x	x	x			x	LGDYLIDQGL	cytoplasm	Cytoskeleton/division
23	A0A2C9U1I9	Protein disulfide-isomerase	971.63	55.64	13.9 - 15.11	5.4	3		x	x	x	x	x	IVIVGVFPK	ER	Protein related
23	A0A2C9WI18	Pyr_redox_2 domain-containing protein	1844.03	54.37	12.57 - 13.99	12	4		x	x		x	x	TNIPGIFAIGDVAAFPLK	cytoplasm	Stress related
23	A0A2C9WA12	Reticulon-like protein	1275.71	28.77	14.58 - 15.12	18.6	5	x	x	x	x	x	x	VEINQAFSVLR	ER (membrane)	Stress related
23	A0A2C9UCU3	Ribosomal_L14e domain-containing protein	1382.80	15.58	10.99 - 20.5	60.4	13	x	x	x	x	x	x	LVVIVDVIDQNR	cytoplasm	translation
23	A0A2C9VVU2	Ribosomal_L18_c domain-containing protein	955.59	23.95	12.1 - 13.64	19.9	5	x	x		x	x		ALLDVGLVR	cytoplasm	translation
23	A0A2C9VFJ3	Ribosomal_L18e/L15P domain-containing protein	1357.77	20.97	12.52 - 15.44	49.1	11	x	x	x	x	x	x	IAVVVGTVTDDIR	cytoplasm	translation
23	A0A199UA18	Ribosomal_L2_C domain-containing protein	1002.52	28.23	12.17 - 15.47	31.4	9	x	x	x	x	x	x	AMVGQVAGGGR	cytoplasm	translation
23	A0A251JVX4	Ribosomal_L23eN domain-containing protein	1418.72	17.33	15.1	50	9			x			x	LTPDFDALDVANK	cytoplasm	translation
23	A0A2C9WBE6	Ribosomal_L7Ae domain-containing protein	1177.67	29.27	12.97 - 18.62	44.1	15	x	x	x			x	KMEVPYAIVK	cytoplasm	translation
23	A0A251J9D7	Ribosomal_S10 domain-containing protein	1332.74	13.73	15 - 16.19	34.9	5	x	x	x	x	x	x	VIDLFSSPEVVK	cytoplasm	translation
23	A0A2C9VS14	Ribosomal_S13_N domain-containing protein	1916.03	17.16	11.73	33.7	5		x			x		AHGLAPEIPEDLYHLIK	cytoplasm/nucleus	translation

Fraction	Database code	Protein name	Protein MW	Protein size (kDa)	Score	Coverage (%)	No. of matched peptides	Spectrum Mill			Mascot			Peptide sequence	Cellular compartment	Biological function
								L	M	H	L	M	H			
23	A0A2C9V9X4	Ribosomal_S7 domain-containing protein	1631.83	23.22	18.76 - 24.28	49.2	10	x	x	x	x	x	x	TIAECLADELINAAK	cytoplasm	translation
23	A0A2C9VM73	Ribulose bisphosphate carboxylase small chain	2038.07	19.58	12.47 - 16.14	26.9	4	x	x	x		x	x	KFETLSYLPPLSDESIAK	plastid	Carbohydrate metabolism
23	A0A2C9U6Q7	RRM domain-containing protein	1232.60	16.36	15.74 - 17.12	23.6	5	x	x	x	x	x	x	GFGFVTFSNEK	unknown	transcription
23	A0A2C9UJQ2	S4 RNA-binding domain-containing protein	1047.58	23.06	14.97 - 16.49	22.3	8	x	x		x	x	x	IFEGEALLR	cytoplasm	Translation
23	A0A2C9VRD2	S5 DRBM domain-containing protein	1229.66	29.62	13.43 - 18.7	43.4	13	x	x	x	x	x	x	VLIEDAEKIEA	cytoplasm	translation
23	A0A2C9VZB5	SAM domain-containing protein	1120.68	27.83	14.26 - 18.33	22.3	7	x	x		x	x		LLILDHIQR	organelle membrane	protein insertion into mitochondrial inner membrane [GO:0045039]; protein targeting to chloroplast [GO:0045036]
23	A0A2C9WHT3	SHSP domain-containing protein	1889.94	26.17	21.9 - 25.25	63.6	29	x	x	x	x	x	x	APWDIKEDENEFKLR	unknown	Stress related
23	B3SRP3	Starch synthase, chloroplastic/amyloplastic	1948.14	83.80	18.46	3	2		x			x		ELGLPIRPDVPVIGFIGR	amyloplast/chloroplast	Carbohydrate metabolism
23	A0A076NB98	Sucrose synthase	2286.21	92.59	22.15 - 23.03	54	44	x	x	x	x	x	x	LLLDLLEAPDPCTLETFLGR	plastid	Carbohydrate metabolism
23	A0A2C9VLE6	Superoxide dismutase	1627.89	25.87	19.42 - 22.47	39	9	x	x	x	x	x	x	LVVETTANQDPLVTK	mitochondria	Stress related
23	A0A2C9UDR8	TCTP domain-containing protein	1063.58	19.04	14.53 - 16.22	14.2	3	x	x	x			x	VVDIVDTFR	cytoplasm	Cell proliferation and differentiation
23	A0A2C9U850	Thioredoxin domain-containing protein	774.37	14.02	12.25 - 15.15	29.6	4	x	x	x			x	DGAQIDR	cytoplasm/nucleus	Stress related
23	A0A2C9VVI6	TRANSKETOLASE_1 domain-containing protein	2057.13	80.58	10.39 - 12.07	11.3	7		x	x		x	x	VLPGLLGGSADLASSNMTLLK	plastid	Carbohydrate metabolism
23	A0A2C9UI51	Tr-type G domain-containing protein	2257.20	94.17	17.68 - 18.65	26.4	24	x	x		x	x	x	STLTDSLVAAAGIIAQEVAGDVR	organelle membrane	translation
23	A0A2C9UFT6	Tubulin alpha chain	1701.91	49.70	18.47 - 19.11	19.3	7	x	x	x		x		AVFVDLEPTVIDEVR	plastid	Cytoskeleton/division
23	A0A2C9UL58	Tubulin beta chain	1215.58	49.84	16.06 - 20.34	21.3	9	x	x	x	x	x		VSEQFTAMFR	plastid	Cytoskeleton/division
23	A0A251JQ24	Uncharacterized protein	1855.93	41.70	20.43 - 23.79	65.2	27	x	x	x	x	x	x	LAYVALDYEQELETAK	cytoplasm	Transport
23	A0A199U950	Uncharacterized protein (Fragment)	3854.87	36.60	20.02 - 21.19	48.3	15	x	x	x	x	x	x	VTPQPGVPPEEAGAAVAAESSTGTWTTVWTDGLTSLDR	chloroplast	Carbohydrate metabolism
23	A0A2C9VGT1	Usp domain-containing protein	1632.85	18.00	24.03 - 24.75	71.4	11	x	x	x	x	x	x	IDIEVLDMLDTISR	unknown	Stress related
23	A0A251LGJ0	UTP--glucose-1-phosphate uridylyltransferase	2081.09	51.50	19.64 - 23.24	64.1	26	x	x	x	x	x	x	VQLLEIAQVPDEHVSEFK	cytoplasm	Carbohydrate metabolism
23	A0A251JTZ2	Vacuolar proton pump subunit B	2772.40	54.43	21.8 - 22.14	39.5	18	x	x	x	x	x		AVVGEEALSSEDLLYLEFLDKFER	organelle membrane	Transport
23	A0A2C9U431	V-type proton ATPase subunit	2136.08	40.67	13.84 - 15.54	8.2	2	x	x	x	x	x	x	LGGATAEIMSDLLAFEADRR	organelle membrane	Transport
23	A0A2C9UZD4	V-type proton ATPase subunit C	1688.93	42.79	11.78 - 14.64	7.7	3	x	x		x	x		VGTLDSLLSLSDDLLK	organelle membrane	Transport
23	A0A2C9V394	V-type proton ATPase subunit G	1103.57	12.05	8.99	18.1	2		x			x		RLEQETEAK	organelle membrane	Transport
23	A0A2C9UNF3	WD_REPEATS_REGION domain-containing protein	1584.87	36.08	13.56 - 14.71	9.7	3	x	x			x		DGVILLWDLAEGKR	cytoplasm/nucleus	Protein related
24	A0A2C9WFF9	14_3_3 domain-containing protein	3313.65	28.50	7.12 - 21.21	69.5	19	x	x	x			x	QAFEEAIAELDTLGEESYKDSTLIMQLLR	cytoplasm	Transport
24	Q1AP39	14-3-3 protein	1788.96	29.83	7.12 - 19.32	47.7	16	x	x	x			x	SAQDIALADLAPTHPIR	cytoplasm	Transport
24	A0A251K8D8	40S ribosomal protein S24	1080.57	15.68	13.02	40.1	7			x			x	DPNTIFVFK	cytoplasm	translation
24	A0A2C9UZC1	40S ribosomal protein S25	1114.65	12.05	15.61	20.3	3		x		x	x		LITPSILSDR	cytoplasm	translation
24	A0A2C9VGW5	40S ribosomal protein S4	1215.71	29.89	17.1 - 17.52	56.2	17		x	x		x	x	TIRYPDPLIK	cytoplasm	translation
24	A0A2C9U2M9	40S ribosomal protein S6	1372.74	28.32	12.19	24.8	9		x			x		KLEIDDDQKLR	cytoplasm	translation
24	A0A2C9UTK8	40S ribosomal protein S7	2349.27	22.09	12.73 - 14.91	24.6	4		x	x			x	TLTAVHEAMLEDIVLPAEIVGK	cytoplasm	translation
24	A0A2C9UDR1	40S ribosomal protein S8	1718.91	24.86	19.35 - 19.67	22.3	6		x	x		x	x	ILDVVYNASNNELVR	cytoplasm	translation
24	A0A2C9V405	60S acidic ribosomal protein P0	1045.59	34.15	11.04 - 19.48	28.1	7		x	x		x		VGSSEAALLAK	cytoplasm	translation
24	A0A2C9VE31	60S ribosomal protein L12	1343.72	17.78	16.22 - 22.43	54.8	8	x	x	x		x	x	VTGGEVGAASSLAPK	cytoplasm	translation
24	A0A2C9VGW4	60S ribosomal protein L13	1214.65	23.69	14.79 - 15.45	34.9	7		x	x			x	SLEGLQANVQR	cytoplasm	translation
24	A0A2C9VSW8	60S ribosomal protein L36	1110.55	12.38	12.67	31.8	5			x			x	EVAGFAPYEK	cytoplasm	translation
24	A0A2C9V6B9	60S ribosomal protein L6	2032.99	25.67	10.26 - 12.42	38	12		x	x			x	VDISGVNVDKFDDTYFAK	cytoplasm	translation
24	A0A2C9VXB8	Aamy domain-containing protein	1422.67	96.69	17.2 - 21.83	55.9	54	x	x	x	x	x		VALDSDAWEFGGR	cytoplasm/plastid	Carbohydrate metabolism
24	A0A2C9WDD9	Alpha-1,4 glucan phosphorylase	1556.68	107.53	14.99 - 24.11	59.3	61	x	x	x	x	x		FADNEDFQTQWR	cytoplasm	Carbohydrate metabolism
24	Q52QX1	Ascorbate peroxidase APX2	2094.88	27.67	24.49 - 25.4	60.4	16		x	x		x	x	YAADEEAFFADYAESHMK	chloroplast	Stress related
24	A0A2C9UKP7	ATP synthase subunit beta	2269.15	60.46	14.66 - 19.38	37	15		x	x		x		FDEGLPPILTSLEVMDHSIR	organelle membrane	Transport
24	A0A2C9VVU3	Catalase domain-containing protein	1557.80	57.34	18.39 - 20.9	46.9	22		x	x		x		GPILLEDYHMIEK	cytoplasm/plasma membrane	Stress related
24	A0A2C9U871	Cytochrome c oxidase subunit 5C	1283.66	6.88	7.59	15.6	1			x			x	AKEFYDLLER	organelle membrane	UNK
24	A0A2C9VPN2	CYTOSOL_AP domain-containing protein	1553.88	60.99	13.95 - 17.84	36.7	20		x	x		x	x	SLDILGLGIGPEIEK	cytoplasm/plastid	remove peptides
24	A0A2C9UC96	DUF3700 domain-containing protein	1448.80	25.38	16.28 - 18.7	23.7	5		x	x		x	x	SANEVILVIEAYK	unknown	Nitrogen/sulphur metabolism
24	A0A2C9WM64	EF1_GNE domain-containing protein	1024.54	23.91	13.55 - 17.07	22.7	4		x	x		x	x	AAAPAEAAGPAK	cytoplasm	translation
24	A0A251J2Y7	Elongation factor 1-alpha	914.57	49.38	8.82 - 11.77	21.1	12		x	x		x	x	QTVAVGVIK	organelle membrane	translation
24	A0A2C9VMR2	Eukaryotic translation initiation factor 5A	1869.99	17.59	12.55 - 13.01	34.3	4	x		x			x	DDLRLPTDENLLSQIK	membrane	Stress related
24	A0A2C9UPJ8	FAS1 domain-containing protein	1192.66	34.64	12.95 - 15.22	17.9	5		x	x		x	x	AFSDLLIASGAK	membrane	Membrane protein
24	A0A2C9VN82	Ferritin	1142.65	29.37	18.4 - 21.59	48.4	11		x	x		x	x	LLNLQSVAER	cytoplasm/plastid	Transport
24	A0A2C9U930	Formate dehydrogenase, mitochondrial	2675.32	42.29	21.47	30.3	13			x			x	AAADAGLTVAEVTGSNVVSVAEDELMR	plastid	Stress related
24	A0A2C9VH59	Fructose-bisphosphate aldolase	3071.57	38.59	15.72 - 18.72	58.1	20		x	x		x	x	TVPAAVPAIVFLSGGQSEEEATLNLNAMNK	cytoplasm	Carbohydrate metabolism
24	A0A251IW93	GLOBIN domain-containing protein	1429.70	18.31	12.54 - 18.2	39.5	6		x	x	x	x	x	EAVPELWTPEMK	unknown	Oxygen carrier
24	A0A2C9UU97	Glucose-1-phosphate adenylyltransferase	1786.94	57.33	17.03 - 20.08	46.8	27		x	x		x	x	LIDIPVSNCLNSNISK	chloroplast	Carbohydrate metabolism
24	A0A2C9UHE4	Glucose-6-phosphate 1-dehydrogenase	1196.74	59.16	14.83	8.3	5			x			x	ELVQNLLVLR	unknown	Carbohydrate metabolism
24	A0A2C9U0T4	Glucose-6-phosphate isomerase	2042.10	67.85	18.03	13.1	7			x			x	TSEMSAVGLLPAALQGIDIR	cytoplasm	Carbohydrate metabolism
24	A0A2C9WLE4	Glutamate decarboxylase	927.48	56.51	14.98	19.4	10			x			x	YFEVELK	cytoplasm	Nitrogen/sulphur metabolism
24	A0A2C9UHS4	Glutaredoxin domain-containing protein	1295.79	11.30	14.15 - 18.79	21.4	2	x	x	x	x		x	LIPLLTEAGAVAK	cytoplasm	Stress related
24	A0A2C9WIS4	Glutathione peroxidase	1376.72	18.66	13.56 - 14.87	18.4	3		x	x		x	x	FLVDKDGNVVDR	cytoplasm	Stress related
24	A0A2C9UK69	Glyceraldehyde-3-phosphate dehydrogenase	1498.85	36.86	14.42 - 19.45	71.8	26		x	x			x	VPTVDVSVVDLTVR	cytoplasm/plastid	Carbohydrate metabolism
24	A0A2C9WGI0	HATPase_c domain-containing protein	2039.07	80.02	20.53 - 21.75	22.7	17		x	x			x	ITLFLKEDQLEYLEER	ER	Protein related
24	A0A2C9UAF1	KH type-2 domain-containing protein	1927.00	26.79	20.31 - 20.58	44.8	13		x	x		x	x	FVADGVFFAELNEVLTR	cytoplasm/nucleus	translation
24	A0A2C9UR13	KOW domain-containing protein	1316.72	16.81	15.67 - 15.82	28	6		x	x		x	x	ILMSAPLSTDLR	cytoplasm	translation
24	A0A251KYX0	Late embryogenesis abundant protein 14-A	2463.32	34.83	14.44	35.8	7			x			x	NPNPVPIPLVDINYLIESDGRK	unknown	Stress related
24	A0A2C9UCS2	Malate dehydrogenase	1346.74	35.80	18.22 - 21.67	57.8	16	x	x	x		x	x	MELVDAAFPLLK	plastid	Carbohydrate metabolism
24	A0A2C9UAX3	Malic enzyme	2205.13	65.00	18.14 - 18.15	48.3	28		x	x		x	x	VLVQFEDFANHNAFELLAK	chloroplast	Carbohydrate metabolism
24	A0A2C9VV21	Nucleoside diphosphate kinase	979.48	16.28	12.42 - 17.97	45.9	9	x	x	x	x	x	x	GDYAIDIGR	plastid/cytoplasm	Nucleic acid related
24	A0A251J7N7	Pectin acetylesterase	1557.73	43.32	12.39	26	11			x			x	DVAGGFEIENFFGR	extracellular region	Cytoskeleton/division
24	A0A2C9W531	Peptidase A1 domain-containing protein	1088.65	46.04	15.76 - 16.03	27.6	10		x	x		x	x	LVAPVYTAVR	membrane	Stress-related
24	A0A2C9V0Y6	Peptidyl-prolyl cis-trans isomerase	1624.91	17.91	12.83 - 17.67	46.1	10	x	x	x	x	x	x	HVVFGQVVEGLDVVK	cytoplasm	Protein related
24	A0A2C9UAL9	Peroxiredoxin	1033.59	17.33	16.35 - 18.24	31.4	7	x	x	x	x	x	x	FALLVDDLK	cytoplasm	Stress related
24	A0A2C9VRW1	Phosphoglycerate kinase	1085.66	42.39	15.06	45.1	15		x			x		LSELLGVQVK	plastid	Carbohydrate metabolism
24	A0A2C9UYF6	Phospholipase D	3412.73	91.91	12.91 - 20.58	35	21		x	x		x	x	AYVPVEELLDGEEIDRWVEILDTDKNPVR	organelle membrane	Lipid metabolism
24	A0A2C9WJ97	PKS_ER domain-containing protein	1100.54	38.62	12.44 - 13.42	30.6	8		x	x			x	VVDSGHPDFK	UNK	Lignin biosynthesis
24	A0A199UAY8	Plasma membrane-associated cation-binding protein 1	1164.63	22.46	15.69	15.6	4			x			x	VSTFVVVEER	membrane	Lipid related
24	V9M4W7	Plastid 16-kDa outer membrane protein	1399.77	15.70	14.73 - 21.72	29.4	7	x	x	x	x	x	x	VLAEDAYHVVKR	chloroplast membrane	Transport
24	A0A2C9UQH2	PLAT domain-containing protein	1235.63	19.32	13.79 - 16.11	20.3	4	x	x	x		x	x	DASPYELTAIR	membrane	Signalling
24	A0A2C9VXF7	Pribosyltran domain-containing protein	1439.75	28.44	12.73	14	4		x			x		AFKDTIDLFVDR	cytoplasm	Nucleic acid related
24	A0A2C9UBP1	Profilin	1432.76	14.10	11.75 - 18.01	23.6	3		x	x			x	YMVIQGEPGAVIR	cytoplasm	Cytoskeleton/division
24	A0A2C9WI18	Pyr_redox_2 domain-containing protein	1446.73	54.37	13.9	12	4			x			x	DVADADALISSLEK	cytoplasm	Stress related
24	A0A2C9VR40	Pyruvate kinase	2333.26	63.26	18.96	6.7	3			x			x	GDLGAELPIEEVPLLQEEIIR	cytoplasm	Protein related

Fraction	Database code	Protein name	Protein MW	Protein size (kDa)	Score	Coverage (%)	No. of matched peptides	Spectrum Mill			Mascot			Peptide sequence	Cellular compartment	Biological function
								L	M	H	L	M	H			
24	A0A2C9WA12	Reticulon-like protein	2071.16	28.77	14.6 - 16.83	18.6	5		x	x		x	x	IPEVHIPEESVLQVAAALR	ER (membrane)	Stress related
24	A0A2C9VC63	Ribos_L4_asso_C domain-containing protein	889.46	45.07	13.73	39.9	16			x			x	MVNADLAR	cytoplasm	translation
24	A0A2C9VYA6	Ribosomal protein	1164.59	24.45	13.03 - 17.93	41.2	10		x	x		x	x	EAISTIMNASK	cytoplasm	translation
24	A0A2C9UIG3	Ribosomal protein L15	1756.94	24.21	16.93 - 22.92	49.5	11		x	x		x	x	YFEVILVDPAHNAIR	cytoplasm	translation
24	A0A2C9WBP1	Ribosomal protein L19	1973.94	24.17	13.36 - 15.51	24.7	6		x	x			x	VWLDPNEVNEISMANSR	cytoplasm	Translation
24	A0A2C9UCU3	Ribosomal_L14e domain-containing protein	1382.80	15.58	13.02 - 22.38	60.4	13	x	x	x		x	x	LVVIVDVIDQNR	cytoplasm	translation
24	A0A2C9VVU2	Ribosomal_L18_c domain-containing protein	955.59	23.95	13.13 - 18.74	19.9	5		x	x		x		ALLDVGLVR	cytoplasm	translation
24	A0A2C9VFJ3	Ribosomal_L18e/L15P domain-containing protein	1357.77	20.97	19.21 - 20.02	49.1	11		x	x		x	x	IAVVVGTVTDDIR	cytoplasm	translation
24	A0A199UA18	Ribosomal_L2_C domain-containing protein	1292.70	28.23	18.05 - 18.29	31.4	9		x	x		x	x	GVVTEIIHDPGR	cytoplasm	translation
24	A0A251KIT5	Ribosomal_L23eN domain-containing protein	1788.99	17.45	13.85 - 16.92	49.3	9		x	x		x	x	KIEDNNTLVFIVDIR	cytoplasm	Translation
24	A0A2C9WBE6	Ribosomal_L7Ae domain-containing protein	945.54	29.27	14.27 - 16.61	44.1	15		x	x			x	VVNPLFEK	cytoplasm	translation
24	A0A251J9D7	Ribosomal_S10 domain-containing protein	1332.74	13.73	14.14 - 18.68	34.9	5	x	x	x		x	x	VIDLFSSPEVVK	cytoplasm	translation
24	A0A2C9VTN5	Ribosomal_S17_N domain-containing protein	1057.55	17.85	12.73	38.9	8			x		x	x	ILAGTCHSAK	cytoplasm	translation
24	A0A2C9V9X4	Ribosomal_S7 domain-containing protein	1631.83	23.22	19.99 - 21.74	49.2	10		x	x		x	x	TIAECLADELINAAK	cytoplasm	translation
24	U3R506	Ribulose bisphosphate carboxylase large chain	1465.76	48.86	12.61- 19.34	44.1	21	x	x	x			x	TFQGPPHGIQVER	chloroplast	Carbohydrate metabolism
24	A0A2C9VM73	Ribulose bisphosphate carboxylase small chain	2038.07	19.58	15.14 - 17.08	26.9	4		x	x		x	x	KFETLSYLPPLSDESIAK	plastid	Carbohydrate metabolism
24	A0A2C9U6Q7	RRM domain-containing protein	1232.60	16.36	13.29 - 18.21	23.6	5	x	x	x	x	x	x	GFGFVTFSNEK	unknown	transcription
24	A0A2C9UJQ2	S4 RNA-binding domain-containing protein	1047.58	23.06	13.65 - 14.17	22.3	8		x	x		x	x	IFEGEALLR	cytoplasm	Translation
24	A0A2C9VRD2	S5 DRBM domain-containing protein	1723.89	29.62	15.06 - 18.98	43.4	13		x	x		x	x	SPFQEYTDLLGKPTK	cytoplasm	translation
24	A0A2C9WHT3	SHSP domain-containing protein	1289.74	26.17	19.87 - 25.13	63.6	29	x	x	x	x	x	x	VFVEEDVLVIK	unknown	Stress related
24	A0A076NB98	Sucrose synthase	2286.21	92.59	19.7 - 21.43	54	44		x	x		x	x	LLLDLLEAPDPCTLETFLGR	plastid	Carbohydrate metabolism
24	A0A2C9VLE6	Superoxide dismutase	1627.89	25.87	20.45 - 22.26	39	9		x	x		x	x	LVVETTANQDPLVTK	mitochondria	Stress related
24	A0A2C9UDR8	TCTP domain-containing protein	1063.58	19.04	11.79 - 15.5	14.2	3		x	x			x	VVDIVDTFR	cytoplasm	Cell proliferation and differentiation
24	A0A2C9U850	Thioredoxin domain-containing protein	774.37	14.02	9.51	29.6	4			x			x	DGAQIDR	cytoplasm/nucleus	Stress related
24	A0A2C9UI51	Tr-type G domain-containing protein	2355.16	94.17	19.06	26.4	24			x		x	x	YRVENLYEGPLDDPYATAIR	organelle membrane	translation
24	A0A2C9UFT6	Tubulin alpha chain	1701.91	49.70	14.54 - 16.85	19.3	7		x	x			x	AVFVDLEPTVIDEVR	plastid	Cytoskeleton/division
24	A0A2C9UL58	Tubulin beta chain	1139.69	49.84	18.09	21.3	9			x			x	LAVNLIPFPR	plastid	Cytoskeleton/division
24	A0A2C9UND2	Ubiquitin-like domain-containing protein	981.58	17.70	10.68 - 14.14	37.8	7		x	x			x	LAVLQFYK	cytoplasm/nucleus	translation
24	A0A251JQ24	Uncharacterized protein	1774.90	41.70	15.74 - 25.87	65.2	27	x	x	x		x	x	NYELPDGQVITIGAER	cytoplasm	Transport
24	A0A199U950	Uncharacterized protein (Fragment)	1465.76	36.60	12.61 - 19.34	48.3	15	x	x	x		x	x	TFQGPPHGIQVER	chloroplast	Carbohydrate metabolism
24	A0A2C9VGT1	Usp domain-containing protein	1632.85	18.00	18.98 - 23.62	71.4	11	x	x	x		x	x	IDIEVLDMLDTISR	unknown	Stress related
24	A0A251LGJ0	UTP--glucose-1-phosphate uridylyltransferase	2496.32	51.50	17.29 - 21.08	64.1	26		x	x		x	x	ATSDLLLVQSDLYILEDGYVIR	cytoplasm	Carbohydrate metabolism
24	A0A251JTZ2	Vacuolar proton pump subunit B	2772.40	54.43	15.01	39.5	18			x			x	AVVGEEALSSEDLLYLEFLDKFER	organelle membrane	Transport
24	A0A251JTM1	V-type proton ATPase proteolipid subunit	1847.95	16.62	10.95	10.9	1		x			x		SGVGVASMGVMRPELVMK	organelle membrane	Transport
24	A0A2C9UNF3	WD_REPEATS_REGION domain-containing protein	1584.87	36.08	13.51	9.7	3			x			x	DGVILLWDLAEGKR	cytoplasm/nucleus	Protein related
25	A0A2C9WFF9	14_3_3 domain-containing protein	1686.89	28.50	17.7 - 21.62	69.5	19	x	x	x		x	x	LVLGSTPSGELTVEER	cytoplasm	Transport
25	Q1AP39	14-3-3 protein	1788.96	29.83	17.82 - 21.09	47.7	16	x	x	x	x	x	x	SAQDIALADLAPTHPIR	cytoplasm	Transport
25	A0A2C9V071	40S ribosomal protein S12	909.53	14.75	12.36 - 13.49	30	4	x		x	x			SLAHGGLVR	cytoplasm	translation
25	A0A251K8D8	40S ribosomal protein S24	1586.77	15.68	16.74	40.1	7	x			x		x	STGFGLIYDSVDNAK	cytoplasm	translation
25	A0A2C9UZC1	40S ribosomal protein S25	1114.65	12.05	15.57 - 16.33	20.3	3	x		x	x		x	LITPSILSDR	cytoplasm	translation
25	A0A2C9VGW5	40S ribosomal protein S4	1215.71	29.89	16.02 - 18.72	56.2	17	x	x	x	x	x	x	TIRYPDPLIK	cytoplasm	translation
25	A0A2C9U2M9	40S ribosomal protein S6	1040.65	28.32	11.71	24.8	9	x			x		x	LVTPLTLQR	cytoplasm	translation
25	A0A2C9UTK8	40S ribosomal protein S7	1252.61	22.09	18.18	24.6	4		x			x		DVVFEFPVTEA	cytoplasm	translation
25	A0A2C9UDR1	40S ribosomal protein S8	1718.91	24.86	18.12 - 19.85	22.3	6	x	x	x	x	x	x	ILDVVYNASNNELVR	cytoplasm	translation
25	A0A2C9UIY2	40S ribosomal protein SA	940.58	34.19	13.78	14.1	5		x		x	x		LLILTDPR	cytoplasm	translation
25	A0A2C9V405	60S acidic ribosomal protein P0	3519.91	34.15	15.39 - 20.3	28.1	7	x	x	x	x	x	x	VGLVAPIDVVVPPGNTGLDPSQTSFFQVLNIPTK	cytoplasm	translation
25	A0A2C9VE31	60S ribosomal protein L12	1343.72	17.78	19.8 - 23.59	54.8	8	x	x	x		x	x	VTGGEVGAASSLAPK	cytoplasm	translation
25	A0A2C9VGW4	60S ribosomal protein L13	1214.65	23.69	15.3 - 17.47	34.9	7	x	x	x		x	x	SLEGLQANVQR	cytoplasm	translation
25	A0A2C9VAV6	60S ribosomal protein L27	869.56	15.63	14.88 - 16.02	19.2	4		x	x	x	x	x	AVIILQGR	cytoplasm	translation
25	A0A2C9VSW8	60S ribosomal protein L36	1110.55	12.38	16.44 - 18.63	31.8	5	x		x	x		x	EVAGFAPYEK	cytoplasm	translation
25	A0A2C9V6B9	60S ribosomal protein L6	2032.99	25.67	18.11 - 18.88	38	12		x	x		x		VDISGVNVDKFDDTYFAK	cytoplasm	translation
25	A0A2C9VXB8	Aamy domain-containing protein	1422.67	96.69	20.86 - 22.35	55.9	54	x	x	x			x	VALDSDAWEFGGR	cytoplasm/plastid	Carbohydrate metabolism
25	A0A2C9UDE9	Adenosylhomocysteinase	905.48	53.30	10.59 - 14.98	19.5	9	x	x			x		ATDVMIAGK	cytoplasm	Amino acid synthesis
25	A0A2C9V321	ADF-H domain-containing protein	2542.27	16.02	13.56 - 18.2	43.1	7	x	x				x	ELDGIQVELQATDPTEMGLDVIR	cytoplasm	Cytoskeleton/division
25	A0A2C9VPJ9	Aldedh domain-containing protein	1636.82	54.86	13.78 - 19.02	29.4	10	x	x	x	x	x	x	ELGEWGLENYLSVK	cytoplasm/plastid	Stress related (potentially Carbohydrae metabolism, Carotenoid cleavage or Amino acid synthesis)
25	A0A2C9WDD9	Alpha-1,4 glucan phosphorylase	2863.37	107.53	20.1 - 23.65	59.3	61	x	x	x	x	x	x	QEVGEDNFFLFGAEAHEIAGLREER	cytoplasm	Carbohydrate metabolism
25	A0A2C9U177	Annexin	2171.99	36.03	17.32 - 18.89	62.6	20	x	x	x	x	x	x	DTHGDYEDMLLALIGHKED	cytoplasm	Stress related
25	Q52QX1	Ascorbate peroxidase APX2	2094.88	27.67	22.98 - 28.89	60.4	16	x	x	x	x	x	x	YAADEEAFFADYAESHMK	chloroplast	Stress related
25	A0A251J147	Aspartate aminotransferase	1508.77	50.80	10.16 - 12.6	10.3	5		x	x	x		x	TVGLDFEGMIADIK	plastid	Nitrogen/sulphur metabolism
25	A0A1B4ZAZ5	ATP synthase subunit alpha, beta	1210.68	55.60	5.97 - 19.16	34.7	17	x	x	x	x		x	VVDALGVPIDGR	organelle membrane	Transport
25	Q9SW99	Catalase CAT1	2732.25	57.17	19.49 - 21.47	35.5	19	x	x	x	x			LFIQTMDPADEDKFDFDPLDMTK	cytoplasm/plasma membrane	Stress related
25	A0A2C9VVU3	Catalase domain-containing protein	2732.25	57.34	19.49 - 21.47	46.9	22	x	x	x	x	x	x	LFIQTMDPADEDKFDFDPLDMTK	cytoplasm/plasma membrane	Stress related
25	A0A2C9UBC8	Cytochrome b5 heme-binding domain-containing protein	866.46	10.95	12.64	14	2			x	x		x	VFDVTTGK	membrane	Membrane protein
25	A0A2C9UBZ0	Cytochrome b-c1 complex subunit 7	1230.68	14.71	12.43	9	1	x			x			EALGALPLYQR	mitochondria	Transport
25	A0A2C9UWB4	Cytochrome c domain-containing protein	936.54	12.18	12.78	7.1	1	x			x			TDLIAYLK	mitochondria (membrane)	Transport
25	A0A2C9U871	Cytochrome c oxidase subunit 5C	1283.66	6.88	9.61	15.6	1	x			x			AKEFYDLLER	organelle membrane	UNK
25	A0A2C9VPN2	CYTOSOL_AP domain-containing protein	1537.86	60.99	18.17 - 20.62	36.7	20	x	x	x	x	x	x	ATLGLTQPANIDVPK	cytoplasm/plastid	remove peptides
25	A0A2C9VLK4	DHO_dh domain-containing protein	905.56	46.56	13.16	7	4	x			x			AVHPIALGK	cytoplasm	Amino acid synthesis
25	A0A2C9UC96	DUF3700 domain-containing protein	1448.80	25.38	15.42 - 17.69	23.7	5	x	x	x	x	x	x	SANEVILVIEAYK	unknown	Nitrogen/sulphur metabolism
25	A0A2C9WM64	EF1_GNE domain-containing protein	1374.71	23.91	17 - 17.32	22.7	4		x	x		x	x	SVEMPGLLWGASK	cytoplasm	translation
25	A0A2C9VMR2	Eukaryotic translation initiation factor 5A	2659.30	17.59	11.39 - 17.15	34.3	4	x		x	x		x	TDYQLIDISEDGFVSLLTETGNTK	membrane	Stress related
25	A0A2C9WKS1	FabA domain-containing protein	1006.57	25.51	14.17	3.4	1			x		x	x	FPFLLVDR	cytoplasm	Lipid metabolism
25	A0A2C9UPJ8	FAS1 domain-containing protein	1192.66	34.64	13.55 - 15.62	17.9	5	x	x	x	x	x	x	AFSDLLIASGAK	membrane	Membrane protein
25	A0A2C9VN82	Ferritin	1142.65	29.37	14.57 - 17.81	48.4	11	x	x	x		x	x	LLNLQSVAER	cytoplasm/plastid	Transport
25	A0A2C9U930	Formate dehydrogenase, mitochondrial	2675.32	42.29	11.94 - 17.02	30.3	13	x	x	x	x	x	x	AAADAGLTVAEVTGSNVVSVAEDELMR	plastid	Stress related
25	A0A2C9VH59	Fructose-bisphosphate aldolase	1772.00	38.59	22.2 - 24.54	58.1	20	x	x	x	x	x	x	VAPEVIAEYTVLALQR	cytoplasm	Carbohydrate metabolism
25	A0A251IW93	GLOBIN domain-containing protein	1391.74	18.31	14.87 - 23.74	39.5	6	x	x	x	x	x	x	VFTEEQEALVVK	unknown	Oxygen carrier
25	A0A2C9UU97	Glucose-1-phosphate adenylyltransferase	1786.94	57.33	16.25 - 22.78	46.8	27	x	x	x	x	x	x	LIDIPVSNCLNSNISK	chloroplast	Carbohydrate metabolism
25	A0A2C9UHE4	Glucose-6-phosphate 1-dehydrogenase	1196.74	59.16	14.81 - 18.48	8.3	5	x	x	x	x	x	x	ELVQNLLVLR	unknown	Carbohydrate metabolism
25	A0A2C9U0T4	Glucose-6-phosphate isomerase	2042.10	67.85	12.51 - 17.96	13.1	7	x	x	x	x		x	TSEMSAVGLLPAALQGIDIR	cytoplasm	Carbohydrate metabolism
25	A0A2C9WLE4	Glutamate decarboxylase	927.48	56.51	11.2 - 14.27	19.4	10		x	x			x	YFEVELK	cytoplasm	Nitrogen/sulphur metabolism
25	A0A2C9UHS4	Glutaredoxin domain-containing protein	1295.79	11.30	15.99 - 19.07	21.4	2	x		x	x		x	LIPLLTEAGAVAK	cytoplasm	Stress related

Fraction	Database code	Protein name	Protein MW	Protein size (kDa)	Score	Coverage (%)	No. of matched peptides	Spectrum Mill			Mascot			Peptide sequence	Cellular compartment	Biological function
								L	M	H	L	M	H			
25	A0A2C9WIS4	Glutathione peroxidase	1376.72	18.66	16.39	18.4	3			x			x	FLVDKDGNVVDR	cytoplasm	Stress related
25	A0A2C9WIS4	Glutathione peroxidase	1376.72	18.66	13.05	18.4	3			x			x	FLVDKDGNVVDR	cytoplasm	Stress related
25	A0A2C9UK69	Glyceraldehyde-3-phosphate dehydrogenase	3025.43	36.86	16.54 - 19.63	71.8	26	x	x	x	x	x	x	NPEEIPWAETGAEYIVESTGVFTDKDK	cytoplasm/plastid	Carbohydrate metabolism
25	A0A2C9WGI0	HATPase_c domain-containing protein	2039.07	80.02	18.33 - 23.69	22.7	17	x	x	x			x	ITLFLKEDQLEYLEER	ER	Protein related
25	A0A199UC09	Histone H2B	774.41	15.40	11.32	45.3	7	x			x	x		LAQEASR	nucleus/plastid	Nucleic acid related
25	A0A251LEJ6	Histone H4	1180.62	11.41	17.74	50.4	5	x			x	x	x	ISGLIYEETR	nucleus	Nucleic acid related
25	A0A251K3C9	HP domain-containing protein	1060.64	101.91	13.45	2.3	3	x			x		x	ALEVIQFLK	unknown	Cytoskeleton/division
25	A0A251LG18	Isocitrate dehydrogenase [NADP]	2069.01	46.43	13.95	7	2	x			x			DQYLNTEEFIDAVAAELK	mitochondria	Carbohydrate metabolism
25	A0A2C9UAF1	KH type-2 domain-containing protein	1423.67	26.79	16.16 - 23.07	44.8	13	x	x	x		x	x	ELAEDGYSGVEVR	cytoplasm/nucleus	translation
25	A0A2C9UR13	KOW domain-containing protein	1316.72	16.81	14.16 - 15.13	28	6	x		x	x	x	x	ILMSAPLSTDLR	cytoplasm	translation
25	A0A2C9U4J9	Lactoylglutathione lyase	1543.83	32.61	11.13 - 21.81	40.8	12	x		x	x		x	SAEVVNLVTQELGGK	cytoplasm/plastid	Stress related
25	A0A251KYX0	Late embryogenesis abundant protein 14-A	1328.58	34.83	16.19	35.8	7	x			x			DFGSALWDMMR	unknown	Stress related
25	A0A2C9UCS2	Malate dehydrogenase	1346.74	35.80	17.83 - 20.64	57.8	16	x	x	x	x	x	x	MELVDAAFPLLK	plastid	Carbohydrate metabolism
25	A0A2C9UAX3	Malic enzyme	2714.43	65.00	18.55 - 21.22	48.3	28	x	x	x	x	x	x	LLIDNVEELLPIVYTPTVGEACQK	chloroplast	Carbohydrate metabolism
25	A0A2C9UPS6	NTF2 domain-containing protein	1733.79	13.60	11.34 - 14.89	34.9	3	x		x	x		x	AFVEHYYTTFDANR	cytoplasm	Transport
25	A0A2C9VV21	Nucleoside diphosphate kinase	1539.81	16.28	19.31 - 20.32	45.9	9	x	x	x	x	x	x	IIGATNPGDSAPGTIR	plastid/cytoplasm	Nucleic acid related
25	A0A251J7N7	Pectin acetylesterase	1557.73	43.32	19.08 - 21.85	26	11	x	x	x	x	x	x	DVAGGFEIENFFGR	extracellular region	Cytoskeleton/division
25	A0A2C9WN28	Pectinesterase	1181.61	61.74	13.2	3.2	2			x			x	MLMFIGDGIGK	membrane	Stress-related
25	A0A2C9W531	Peptidase A1 domain-containing protein	2951.52	46.04	17.04 - 17.07	27.6	10	x	x	x	x	x	x	VVDIPPSALAFNPTTGAGTIFDSGTVFTR	membrane	Stress-related
25	A0A2C9VBW3	Peptidase_M24 domain-containing protein	1626.88	43.34	9.72	7.8	3			x			x	AADVIAAANTAAEVALR	unknown	Protein related
25	A0A2C9V0Y6	Peptidyl-prolyl cis-trans isomerase	1624.91	17.91	14 - 16.74	46.1	10	x	x	x	x	x	x	HVVFGQVVEGLDVVK	cytoplasm	Protein related
25	A0A2C9UAL9	Peroxiredoxin	2107.06	17.33	19.08 - 20.94	31.4	7	x	x	x	x	x	x	AANLEQGGEFTVSSVDEILK	cytoplasm	Stress related
25	A0A2C9V0F7	PfkB domain-containing protein	1615.82	35.29	19.06	9.9	3	x			x			LGDDEFGHMLAGILK	cytoplasm	Carbohydrate metabolism
25	A0A2C9VRW1	Phosphoglycerate kinase	1573.84	42.39	13.65 - 16.34	45.1	15	x	x		x			GVTTIIGGGDSVAAVEK	plastid	Carbohydrate metabolism
25	A0A2C9UYF6	Phospholipase D	1146.60	91.91	13.97 - 17.91	35	21	x		x	x			VLMLVWDDR	organelle membrane	Lipid metabolism
25	A0A2C9WJ97	PKS_ER domain-containing protein	1192.67	38.62	12.27 - 17.25	30.6	8	x	x	x	x	x	x	FLDFVVPSIR	UNK	Lignin biosynthesis
25	A0A199UAY8	Plasma membrane-associated cation-binding protein 1	1164.63	22.46	16.48	15.6	4			x			x	VSTFVVVEER	membrane	Lipid related
25	V9M4W7	Plastid 16-kDa outer membrane protein	788.46	15.70	12.68 - 17.85	29.4	7	x	x	x	x	x	x	IGTVAATR	chloroplast membrane	Transport
25	A0A2C9UQH2	PLAT domain-containing protein	1235.63	19.32	13.26 - 21.17	20.3	4	x	x	x	x	x	x	DASPYELTAIR	membrane	Signalling
25	A0A2C9UBP1	Profilin	1432.76	14.10	11.88 - 16.74	23.6	3	x	x	x			x	YMVIQGEPGAVIR	cytoplasm	Cytoskeleton/division
25	A0A2C9UZL0	Proteasome subunit alpha type	1101.62	27.13	13.08 - 16.84	20	5	x	x	x	x	x	x	ALLEVVESGGK	cytoplasm/nucleus	Protein related
25	A0A2C9W5C7	Proteasome subunit beta	1188.66	24.95	14.25 - 16.22	11.6	3		x	x		x	x	TVIINSEGVTR	cytoplasm/nucleus	Protein related
25	A0A2C9U1I9	Protein disulfide-isomerase	971.63	55.64	18.08	5.4	3	x			x			IVIVGVFPK	ER	Protein related
25	A0A2C9VCZ9	Pyr_redox_2 domain-containing protein	1878.99	47.18	14.35 - 16.08	15.6	7	x		x	x		x	TSVPDVYAVGDVATFPLK	cytoplasm	Stress related
25	A0A2C9VCP1	Pyruvate kinase	2485.28	57.33	13.57 - 13.8	10.4	3	x		x			x	AEATDVANAVLDGSDAILLGAETLR	chloroplast stroma	Carbohydrate metabolism
25	A0A2C9WA12	Reticulon-like protein	2071.16	28.77	15.13 - 16.78	18.6	5		x	x		x	x	IPEVHIPEESVLQVAAALR	ER (membrane)	Stress related
25	A0A2C9VC63	Ribos_L4_asso_C domain-containing protein	889.46	45.07	12.5 - 17.26	39.9	16		x	x	x		x	MVNADLAR	cytoplasm	translation
25	A0A2C9VYA6	Ribosomal protein	1505.82	24.45	17.22 - 19.48	41.2	10		x	x	x	x		NFTETIELQIGLK	cytoplasm	translation
25	A0A2C9UIG3	Ribosomal protein L15	1756.94	24.21	22.05 - 23.95	49.5	11		x	x		x	x	YFEVILVDPAHNAIR	cytoplasm	translation
25	A0A2C9WBP1	Ribosomal protein L19	1973.94	24.17	13.32 - 18.71	24.7	6	x	x	x			x	VWLDPNEVNEISMANSR	cytoplasm	Translation
25	A0A2C9UCU3	Ribosomal_L14e domain-containing protein	1382.80	15.58	16.25 - 22	60.4	13	x	x	x	x	x	x	LVVIVDVIDQNR	cytoplasm	translation
25	A0A2C9VVU2	Ribosomal_L18_c domain-containing protein	955.59	23.95	10.92 - 14.19	19.9	5	x		x		x		ALLDVGLVR	cytoplasm	translation
25	A0A2C9UPC7	Ribosomal_L18e/L15P domain-containing protein	1264.76	16.47	14.73 - 16.93	49.3	7	x	x	x			x	GALPENKPIVVK	cytoplasm	Translation
25	A0A2C9VFJ3	Ribosomal_L18e/L15P domain-containing protein	1550.76	20.97	14.02 - 23.14	49.1	11	x	x	x	x	x	x	AGGECLTFDQLALR	cytoplasm	translation
25	A0A199UA18	Ribosomal_L2_C domain-containing protein	1002.52	28.23	16.52 - 20.74	31.4	9	x	x	x	x	x	x	AMVGQVAGGGR	cytoplasm	translation
25	A0A251KIT5	Ribosomal_L23eN domain-containing protein	1788.99	17.45	15.26 - 18.06	49.3	9	x	x	x	x	x	x	KIEDNNTLVFIVDIR	cytoplasm	Translation
25	A0A2C9WBE6	Ribosomal_L7Ae domain-containing protein	1177.67	29.27	14.55 - 16.68	44.1	15	x	x	x		x	x	KMEVPYAIVK	cytoplasm	translation
25	A0A251J9D7	Ribosomal_S10 domain-containing protein	1332.74	13.73	16.7 - 18.87	34.9	5	x	x	x	x	x	x	VIDLFSSPEVVK	cytoplasm	translation
25	A0A2C9VS14	Ribosomal_S13_N domain-containing protein	1916.03	17.16	14.85 - 16.29	33.7	5	x	x		x			AHGLAPEIPEDLYHLIK	cytoplasm/nucleus	translation
25	A0A2C9V9X4	Ribosomal_S7 domain-containing protein	1631.83	23.22	13.21 - 24.68	49.2	10	x	x	x	x	x	x	TIAECLADELINAAK	cytoplasm	translation
25	A0A2C9VM73	Ribulose bisphosphate carboxylase small chain	2038.07	19.58	15.45 - 16.93	26.9	4	x		x	x		x	KFETLSYLPPLSDESIAK	plastid	Carbohydrate metabolism
25	A0A2C9U6Q7	RRM domain-containing protein	1232.60	16.36	16.36 - 18.71	23.6	5	x	x	x	x	x	x	GFGFVTFSNEK	unknown	transcription
25	A0A2C9UJQ2	S4 RNA-binding domain-containing protein	1047.58	23.06	10.99 - 15.57	22.3	8	x	x	x	x	x	x	IFEGEALLR	cytoplasm	Translation
25	A0A2C9VRD2	S5 DRBM domain-containing protein	1229.66	29.62	11.74 - 16.05	43.4	13	x	x	x	x	x		VLIEDAEKIEA	cytoplasm	translation
25	A0A2C9WHT3	SHSP domain-containing protein	1336.66	26.17	22.51 - 23.76	63.6	29	x	x	x	x	x	x	IFEDAMTLPGSR	unknown	Stress related
25	A0A076NB98	Sucrose synthase	2286.21	92.59	17.94 - 21.89	54	44	x	x	x	x	x	x	LLLDLLEAPDPCTLETFLGR	plastid	Carbohydrate metabolism
25	A0A2C9VLE6	Superoxide dismutase	1627.89	25.87	13.57 - 20.24	39	9	x	x	x		x	x	LVVETTANQDPLVTK	mitochondria	Stress related
25	A0A2C9UDR8	TCTP domain-containing protein	1063.58	19.04	12.74 - 14.92	14.2	3	x		x			x	VVDIVDTFR	cytoplasm	Cell proliferation and differentiation
25	A0A2C9UDR8	TCTP domain-containing protein	1063.58	19.04	10.69 - 12.39	14.2	3	x		x			x	VVDIVDTFR	cytoplasm	Cell proliferation and differentiation
25	A0A2C9VD17	Thioredoxin domain-containing protein	1762.96	29.38	13.99 - 14.02	22.3	8	x		x	x	x	x	SGGLGDLKYPLISDVTK	plastid	Stress related
25	A0A2C9VWH6	Thioredoxin domain-containing protein	1137.72	16.41	10.22 - 18.53	12.3	2	x	x	x	x	x	x	LIAPILADLAK	plastid	Stress related
25	A0A2C9VVI6	TRANSKETOLASE_1 domain-containing protein	2057.13	80.58	13.47 - 15.54	11.3	7	x		x	x			VLPGLLGGSADLASSNMTLLK	plastid	Carbohydrate metabolism
25	A0A2C9UI51	Tr-type G domain-containing protein	2355.16	94.17	17.66 - 24.21	26.4	24	x	x	x	x	x	x	YRVENLYEGPLDDPYATAIR	organelle membrane	translation
25	A0A2C9UFT6	Tubulin alpha chain	1701.91	49.70	13.32 - 18.33	19.3	7	x	x	x			x	AVFVDLEPTVIDEVR	plastid	Cytoskeleton/division
25	A0A2C9UL58	Tubulin beta chain	1139.69	49.84	15.25 - 16.88	21.3	9	x	x	x			x	LAVNLIPFPR	plastid	Cytoskeleton/division
25	A0A2C9UND2	Ubiquitin-like domain-containing protein	981.58	17.70	12.17 - 13.59	37.8	7	x		x			x	LAVLQFYK	cytoplasm/nucleus	translation
25	A0A251JQ24	Uncharacterized protein	1774.90	41.70	19.95 - 22.53	65.2	27	x	x	x	x	x	x	NYELPDGQVITIGAER	cytoplasm	Transport
25	A0A199U950	Uncharacterized protein (Fragment)	1465.76	36.60	13.49 - 18.15	48.3	15	x	x	x	x	x		TFQGPPHGIQVER	chloroplast	Carbohydrate metabolism
25	A0A2C9VGT1	Usp domain-containing protein	1632.85	18.00	23.57 - 24.86	71.4	11	x	x	x	x	x	x	IDIEVLDMLDTISR	unknown	Stress related
25	A0A251LGJ0	UTP--glucose-1-phosphate uridylyltransferase	2938.49	51.50	19.73 - 21.7	64.1	26	x	x	x	x	x	x	IQTPTDEVVVPYDSLAPIPDDPAETKK	cytoplasm	Carbohydrate metabolism
29	A0A2C9WFF9	14_3_3 domain-containing protein	1772.97	28.50	15.06 - 16.46	69.5	19	x	x			x		AAQDIALADLAPTHPIR	cytoplasm	Transport
29	A0A2C9VXB8	Aamy domain-containing protein	1422.67	96.69	21.74	55.9	54		x			x		VALDSDAWEFGGR	cytoplasm/plastid	Carbohydrate metabolism
29	A0A2C9WFG6	Aconitate hydratase	1376.72	98.32	15.87	6.5	6		x			x		VVQSNVLPDMFK	cytoplasm/mitochondrion	Carbohydrate metabolism
29	A0A2C9UDE9	Adenosylhomocysteinase	2501.29	53.30	17.73	19.5	9		x			x		TGQVPDPSSTDNAEFQIVLTIIR	cytoplasm	Amino acid synthesis
29	A0A2C9VPJ9	Aldedh domain-containing protein	1636.82	54.86	20.65	29.4	10		x			x		ELGEWGLENYLSVK	cytoplasm/plastid	Stress related (potentially Carbohydrae metabolism, Carotenoid cleavage or Amino acid synthesis)
29	A0A2C9WDD9	Alpha-1,4 glucan phosphorylase	2502.12	107.53	14.73 - 21.25	59.3	61	x	x	x	x	x	x	DGQEEVAEDWLEMGNPWEIVR	cytoplasm	Carbohydrate metabolism
29	A0A2C9U177	Annexin	3097.43	36.03	16.42 - 20.46	62.6	20	x	x	x	x	x	x	ETYYEAYGEDLLKELDRELSNDFER	cytoplasm	Stress related
29	A0A2C9VPN2	CYTOSOL_AP domain-containing protein	1294.65	60.99	17.34 - 21.36	36.7	20	x	x	x	x	x	x	FDMGGSAAVLGAAK	cytoplasm/plastid	remove peptides
29	A0A2C9UC96	DUF3700 domain-containing protein	1448.80	25.38	13.19 - 18.14	23.7	5	x	x		x	x		SANEVILVIEAYK	unknown	Nitrogen/sulphur metabolism
29	A0A2C9WM64	EF1_GNE domain-containing protein	1374.71	23.91	12.32	22.7	4		x			x		SVEMPGLLWGASK	cytoplasm	translation
29	A0A2C9UPJ8	FAS1 domain-containing protein	1192.66	34.64	13.12 - 18.29	17.9	5	x	x	x	x	x	x	AFSDLLIASGAK	membrane	Membrane protein
29	A0A2C9VN82	Ferritin	1142.65	29.37	15.14 - 17.18	48.4	11	x	x	x	x	x	x	LLNLQSVAER	cytoplasm/plastid	Transport

Fraction	Database code	Protein name	Protein MW	Protein size (kDa)	Score	Coverage (%)	No. of matched peptides	Spectrum Mill			Mascot			Peptide sequence	Cellular compartment	Biological function
								L	M	H	L	M	H			
29	A0A2C9VH59	Fructose-bisphosphate aldolase	1772.00	38.59	17.51 - 24.27	58.1	20	x	x	x	x	x	x	VAPEVIAEYTVLALQR	cytoplasm	Carbohydrate metabolism
29	A0A251IW93	GLOBIN domain-containing protein	1391.74	18.31	12.55 - 20.26	39.5	6	x		x	x			VFTEEQEALVVK	unknown	Oxygen carrier
29	A0A2C9UU97	Glucose-1-phosphate adenylyltransferase	2366.14	57.33	14.89 - 15.97	46.8	27	x	x	x	x	x	x	DKFPGANDFGSEVIPGATSIGMR	chloroplast	Carbohydrate metabolism
29	A0A2C9UHE4	Glucose-6-phosphate 1-dehydrogenase	1196.74	59.16	19.33	8.3	5		x			x		ELVQNLLVLR	unknown	Carbohydrate metabolism
29	A0A2C9U0T4	Glucose-6-phosphate isomerase	2042.10	67.85	18.5	13.1	7		x			x		TSEMSAVGLLPAALQGIDIR	cytoplasm	Carbohydrate metabolism
29	A0A2C9UK69	Glyceraldehyde-3-phosphate dehydrogenase	1498.85	36.86	9.95 - 18.47	71.8	26	x	x	x	x	x		VPTVDVSVVDLTVR	cytoplasm/plastid	Carbohydrate metabolism
29	A0A251LG18	Isocitrate dehydrogenase [NADP]	1251.67	46.43	17.89	7	2		x			x		LIDDMVAYALK	mitochondria	Carbohydrate metabolism
29	A0A2C9U4J9	Lactoylglutathione lyase	1543.83	32.61	18 - 20.33	40.8	12	x	x	x	x	x	x	SAEVVNLVTQELGGK	cytoplasm/plastid	Stress related
29	A0A2C9UCS2	Malate dehydrogenase	1650.00	35.80	18.34 - 20.32	57.8	16	x	x	x	x	x	x	VLVVANPANTNALILK	plastid	Carbohydrate metabolism
29	A0A2C9UAX3	Malic enzyme	2205.13	65.00	13.18 - 21.76	48.3	28	x	x	x	x	x	x	VLVQFEDFANHNAFELLAK	chloroplast	Carbohydrate metabolism
29	A0A2C9VBG8	NAD(P)-bd_dom domain-containing protein	1347.69	27.47	16.72	20.7	3		x			x		IGGAEDVFVGDIR	plastid membrane	Stress related
29	A0A2C9VV21	Nucleoside diphosphate kinase	1370.67	16.28	16.64 - 18.16	45.9	9	x		x	x			NVIHGSDSVESAR	plastid/cytoplasm	Nucleic acid related
29	A0A251J7N7	Pectin acetylesterase	1557.73	43.32	23.35 - 24.52	26	11	x	x		x	x		DVAGGFEIENFFGR	extracellular region	Cytoskeleton/division
29	A0A2C9W531	Peptidase A1 domain-containing protein	1088.65	46.04	12.86 - 16.52	27.6	10	x	x	x	x	x	x	LVAPVYTAVR	membrane	Stress-related
29	A0A2C9WF26	Peroxidase	1759.89	38.63	18.2 - 21.12	27.3	11	x	x		x	x		DSVVLTGGPDYDVPLGR	extracellular region	Stress related
29	A0A2C9UAL9	Peroxiredoxin	1033.59	17.33	18.94 - 20.11	31.4	7	x		x	x			FALLVDDLK	cytoplasm	Stress related
29	A0A2C9WJ97	PKS_ER domain-containing protein	1192.67	38.62	13.75 - 19.52	30.6	8	x	x		x	x		FLDFVVPSIR	UNK	Lignin biosynthesis
29	A0A2C9UZL0	Proteasome subunit alpha type	1101.62	27.13	14.06 - 17.76	20	5	x	x	x	x	x	x	ALLEVVESGGK	cytoplasm/nucleus	Protein related
29	A0A2C9W5C7	Proteasome subunit beta	1188.66	24.95	13.3 - 13.58	11.6	3		x	x		x	x	TVIINSEGVTR	cytoplasm/nucleus	Protein related
29	A0A2C9V8A9	Pyr_redox_2 domain-containing protein (Fragment)	1878.99	41.03	15.88 - 17.3	14.5	5	x	x		x	x		TSVPDVYAVGDVATFPLK	cytoplasm	Stress related
29	A0A2C9VCP1	Pyruvate kinase	2683.36	57.33	17.04	10.4	3		x			x		IENAEGLTHFDEILQEADGIILSR	chloroplast stroma	Carbohydrate metabolism
29	A0A2C9WHT3	SHSP domain-containing protein	1289.74	26.17	22.55 - 23.06	63.6	29	x	x	x	x	x	x	VFVEEDVLVIK	unknown	Stress related
29	A0A2C9UDR8	TCTP domain-containing protein	1063.58	19.04	12.25 - 14.19	14.2	3	x	x	x		x	x	VVDIVDTFR	cytoplasm	Cell proliferation and differentiation
29	A0A2C9VD17	Thioredoxin domain-containing protein	1762.96	29.38	11.3 - 18.05	22.3	8		x	x		x	x	SGGLGDLKYPLISDVTK	plastid	Stress related
29	A0A2C9UI51	Tr-type G domain-containing protein	1650.84	94.17	9. 99 - 18.67	26.4	24	x	x	x	x	x	x	ILSEEFGWDKDLAK	organelle membrane	translation
29	A0A2C9V8B3	Uncharacterized protein	2252.13	47.86	21.61 - 24.71	66.5	25	x	x	x	x	x	x	SGETEDTFIADLSVGLATGQIK	cytoplasm	Protein related
29	A0A199U950	Uncharacterized protein (Fragment)	1021.53	36.60	11.47 - 19.35	48.3	15		x	x	x	x	x	DTDILAAFR	chloroplast	Carbohydrate metabolism
29	A0A251LGJ0	UTP--glucose-1-phosphate uridylyltransferase	1344.74	51.50	19.55 - 23.15	64.1	26	x	x	x	x	x	x	LVVEDFTPLPSK	cytoplasm	Carbohydrate metabolism
