# Supplementary material for: Carotenoid composition and sequestration in cassava (Manihot esculentum Crantz) roots
Source: PLoS One. 2024 Nov 18;19(11):e0312517. doi: 10.1371/journal.pone.0312517 (PMC11573132; doi:10.1371/journal.pone.0312517)
Supplement: Attachment — Submitted filename: Reviewer report to PLOS ONE editor.pdf [file pone.0312517.s014.pdf]

Dear Editor,

Firstly, I want to express my appreciation for the opportunity to review this paper. I have completed the review of the manuscript titled **" Carotenoid composition and sequestration in cassava (Manihot esculentum Crantz) roots"** and I would like to provide my feedback and recommendation.

The study presented in the manuscript is of high quality and makes a valuable contribution to the field. The authors have conducted a thorough investigation and have addressed the research question with appropriate methodology and analysis. The data analysis is sound, and the findings are well-supported by the provided evidence.

Based on my review, I am pleased to recommend the acceptance of this manuscript for publication in the PLOS ONE Journal.

While I have identified a few minor suggestions for improvement, they do not detract from the overall quality and impact of the study. I have communicated these suggestions directly to the authors in my review, and I am confident that they will address them adequately in the final version.

Thank you for considering my recommendation. Please do not hesitate to reach out to me if you require any further clarification or additional information.

Sincerely,

Dr. Fatma Mohamed Abd El-Mordy (Ph.D)
Department of Pharmacognosy and Medicinal Plants,
Faculty of Pharmacy (Girls),
Al-Azhar University.
Fatmamohammed.pharmg@azhar.edu.eg
